# Cancer stem cell programs S-nitrosylation and deubiquitination of FoxP3 protein to promote regulatory T cell differentiation

**DOI:** 10.3389/fimmu.2026.1961819

**Published:** 2026-09-15

**Authors:** Xiyu Liu, Jiaxin Lei, Xinhang Jiang, Lingyi Li, Longhao Yu, Gengxue Wang, Kaixun Guo, Zhenke Wen, Yan Wang

**Affiliations:** 1 Department of Thoracic Surgery, China-Japan Union Hospital of Jilin University, Jilin University, Changchun, China; 2 Jiangsu Key Laboratory of Infection and Immunity, Institutes of Biology and Medical Sciences, Suzhou Medical College of Soochow University, Suzhou, China; 3 Department of Cardiology, Nanxishan Hospital of Guangxi Zhuang Autonomous Region, Guilin, China; 4 Department of Thoracic Surgery, Central Hospital of Gongzhuling City, Siping, China

**Keywords:** CSCs, deubiquitination, Foxp3, regulatory T cell, S-nitrosylation

## Abstract

**Background:**

Cancer stem cells (CSCs) are critical drivers of tumor progression and therapeutic resistance in non-small cell lung cancer (NSCLC). However, how CSCs remodel the immunosuppressive tumor microenvironment (TME) of NSCLC remains largely unclear.

**Methods:**

Flow cytometry was performed to evaluate the immunomodulatory effects of NSCLC CSCs on T cell differentiation. RNA-sequencing-based metabolic profiling was conducted to identify pivotal metabolic pathways activated in CSCs. Mitochondrial reactive oxygen species (ROS) encapsulated in CSC-derived exosomes were quantified, and the molecular mechanism by which exosomal ROS modulates intracellular nitric oxide (NO) production and FoxP3 post-translational modifications in T cells was further explored. Patient-derived organoids (PDOs) were utilized as a preclinical model to verify the therapeutic potential of glutamine metabolism targeting.

**Results:**

NSCLC CSCs potently induced tumor immunosuppression by promoting regulatory T (Treg) cell differentiation. Mechanistically, hyperactive glutamine metabolism in CSCs substantially increased mitochondrial ROS generation. Exosomal ROS secreted by CSCs was transferred to T cells, thereby elevating intracellular NO synthesis. Increased NO further triggered S-nitrosylation and deubiquitination of FoxP3, which ultimately stabilized FoxP3 expression and facilitated Treg cell differentiation. In NSCLC PDO models, pharmacological inhibition of glutamine metabolism reversed the immunosuppressive T cell phenotype and efficiently suppressed PDO growth.

**Conclusion:**

NSCLC CSCs mediate TME immunosuppression via a glutamine metabolism-dependent regulatory axis. Exosomal ROS-initiated FoxP3 post-translational modification is a novel mechanism underlying CSC-driven Treg differentiation. This study reveals an unreported immune evasion pathway in NSCLC and identifies glutamine metabolism as a viable therapeutic target for overcoming tumor immunosuppression.

## Introduction

Non-small-cell lung cancer (NSCLC) poses a significant challenge in clinical settings, standing as the foremost cause of cancer-related deaths globally (1). Current approaches to treating NSCLC typically encompass a blend of interventions such as surgery, chemotherapy, radiation therapy, targeted therapy, and immunotherapy, tailored to the clinical stage and specific characteristics of the tumor (2). While surgery is commonly employed for early-stage NSCLC, chemotherapy and radiation therapy may serve to reduce tumor size pre-surgery, eradicate residual cancer cells post-surgery, or function as primary treatment for advanced-stage NSCLC (1, 2). Targeted therapy medications are frequently utilized for advanced NSCLC featuring specific mutations, while immunotherapy, including immune checkpoint inhibitors, is frequently employed either independently or in conjunction with other modalities (1, 2). Ongoing clinical trials continue to explore and assess novel treatments for NSCLC, including innovative targeted therapies, immunotherapies, and combination approaches, highlighting the need to investigate key factors influencing anti-tumor immunity within the tumor microenvironment (TME).

CD4^+^ T cells recognize specific antigens presented by antigen-presenting cells and play a pivotal role in coordinating the immune responses (3, 4). Upon activation, they release cytokines and signaling molecules, which stimulate the proliferation and activation of other immune cells, including CD8^+^ cytotoxic T cells and macrophages (3, 5). Notably, CD4^+^ T cells aid CD8^+^ cytotoxic T cells by secreting cytokines such as interleukin-2 (IL-2), IL-12, and interferon-gamma (IFN-γ), thus promoting the expansion and activation of CD8^+^ T cells, augmenting their capacity to directly target and eliminate tumor cells (3, 6). Additionally, within the TME, CD4^+^ T cells can produce IFN-γ and tumor necrosis factor-alpha (TNF-α), exerting anti-tumor effects (3, 7). While regulatory CD4^+^ T (Tregs) cells function to dampen excessive immune responses, their presence in the context of cancer hinders the effectiveness of anti-tumor immunity (3, 8, 9). Consequently, the multifaceted roles of CD4^+^ T cells in tumor immunity underscore their significance as central regulators of the anti-tumor immune response (3). Understanding and leveraging the functions of CD4^+^ T cells are imperative for the development of efficacious immunotherapeutic strategies for cancer treatment.

Cancer cells exhibit heterogeneity, comprising both cancer stem cells (CSCs) and non-CSCs (10–12). CSCs, distinguished by their self-renewal capacity, multilineage differentiation potential, and ability to initiate tumor formation, constitute a distinct subpopulation contributing to tumor initiation, progression, metastasis, and treatment resistance (10, 11). Phenotypically, CSCs express specific stem cell markers include CD44, CD133, ALDH (aldehyde dehydrogenase), and certain embryonic stem cell markers such as OCT4, SOX2, and NANOG. CSCs significantly contribute to tumor heterogeneity by generating a variety of cell types within the tumor and propelling tumor initiation and progression, thereby fueling tumor growth and enabling metastasis (10, 11). Importantly, CSCs frequently evade conventional cancer treatments and play a pivotal role in metastasis by infiltrating blood or lymphatic vessels, surviving in circulation, extravasating into distant organs, and initiating secondary tumor growth (13, 14). Therefore, understanding CSC biology in NSCLC is crucial for developing innovative therapeutic approaches targeting these cells (10, 11). Targeting CSC-specific pathways or vulnerabilities shows potential for improving treatment effectiveness, reducing tumor recurrence, and enhancing patient outcomes (13, 14). However, the potential impact of CSCs on CD4^+^ T cell differentiation remains largely unknown.

In this study, we investigated the role of NSCLC CSCs in regulating CD4^+^ T cell responses, and identified a novel function of CSCs: promoting Treg cell differentiation while suppressing pro-inflammatory T cells. These CSCs showed enriched glutamine metabolism, which increased reactive oxygen species (ROS) levels in T cells. Elevated ROS further enhanced nitric oxide (NO) levels in T cells, thereby promoting S-nitrosylation and deubiquitination of FoxP3 to facilitate Treg cell differentiation. Importantly, inhibition of glutamine metabolism effectively suppressed tumor growth in NSCLC patient-derived organoids (PDOs). Collectively, these findings provide new insights into CSC biology and offer potential avenues for the development of therapeutic strategies for NSCLC management.

## Materials and methods

### Study period

Human sample experiments were performed from December 2020 to April 2026, while animal studies were conducted between January 2023 and December 2025. All data analysis was finalized by May 2026.

### Patients

A total of 33 patients diagnosed with NSCLC and 89 age-matched healthy volunteers were recruited into this study from 2020 to 2026. Patient characteristics were provided in **Table 1**. Following written informed consent, peripheral blood and tumor tissue samples were collected to investigate the effect of CSCs on T cell differentiations. All experimental protocols involving human samples were carried out in adherence with the Declaration of Helsinki and approval by the Ethics Committee of China-Japan Union Hospital of Jilin University (202202005).

**Table 1:** Clinical features of NSCLC patients.

Demographic parameters	NSCLC patients	Healthy donors
No. of subjects	33	89
Sex (F/M)	15/18	43/46
Age (mean ± SEM [years])	51.73 ± 8.11	49.65 ± 5.94
TNM staging (I/II/III)	12/19/2	N/A
Disease duration (mean ± SEM [months])	2.31 ± 1.55	N/A
Histological types		
Adenocarcinoma	29	N/A
Squamous carcinoma	4	N/A

### T cell preparation and cell culture

PBMCs were obtained from either healthy donors or NSCLC patients utilizing the Lymphocyte Isolation Solution (Dakewe Biotech). CD4^+^ T cells were subsequently isolated from PBMCs employing Human CD4^+^ T Cell Isolation Kit (STEMCELL Technologies). Naïve CD4^+^ T cells were isolated from PBMCs utilizing Human Naïve CD4^+^ T Cell Isolation Kit (STEMCELL Technologies) (15, 16).

All cells were cultured in RPMI 1640 medium (Corning) supplemented with 10% FBS (Sigma) and 50 units/mL penicillin/streptomycin (Beyotime).

### Tumor-spheres and adherent cells

Tumor-spheres were enriched using DMEM/F12 medium (Corning, Cat. 10-092-cv) supplemented with 1x B-27 (GIBCO), EGF (20 ng/mL; Novoprotein, Cat. C029), bFGF (20 ng/mL; Novoprotein, C046), insulin (Beyotime, P3376-100IU), and penicillin/streptomycin (50 units/mL, Beyotime, Cat. C0222) as previously described (10, 11). For serial passaging, spheres were harvested after 6 days using a 40 mm cell strainer, dissociated into single cells by continuous pipetting, and then cultured under the same conditions. Sphere formation assays were conducted by quantifying the number of spheres with diameters greater than 40 mm using a CASY Cell Counter (Biocentury).

### CSC-T cell co-culture and T cell differentiations

The CSC-T cell co-culture was established according to previously described method (3). T cells were initially co-cultured with CSCs at a ratio of 4:1 for 12 hours, followed by activation using beads coated with anti-CD3/CD28 beads (at a 1:1 ratio, Gibco). T-cell differentiation was assessed by quantifying intracellular lineage-determining transcription factors and cytokines using flow cytometry (3).

For the *in vivo* differentiation of T cells, CD4^+^ T cells from healthy donor PBMCs were preincubated with or without CSCs at a ratio of 4:1 (T cells: CSCs) for 12 hours. After co-culture, the T cells were reintroduced into the autologous PBMC population (1×10^7^ cells/mouse) and adoptively transferred into 6-week-old immunodeficient NOD-Prkdc^scid^Il2rg^em1^/Smoc (M-NSG) mice (Shanghai Model Organisms) via an intraperitoneal injection without anesthesia. Both male and female mice were used in equal numbers. Sample size was determined by power analysis. A total of 12 mice (6 per group) were used. No inclusion/exclusion criteria were applied; and no animals were excluded from the analysis. Mice were randomly assigned to control and treated groups using a computer-generated random number sequence (www.random.org), and the analysts were not aware of the group allocations. One week later, mice were euthanized with carbon dioxide in their home cage, and the splenic frequencies of human T cells subsets were determined using flow cytometry.

All mice were housed in a standard SPF facility of our institution. The exact number of mice in each group and statistics were clarified in the corresponding figure legends. All procedures were performed in accordance with the ARRIVE guidelines. Experiments were carried out in accordance with our Institutional Animal Care and Use Committee guidelines and approved by the Institutional Ethics Committee of China-Japan Union Hospital of Jilin University (202202072).

### Transfections and reagents

Lentiviral particles for gene overexpression or knockdown in cancer stem cells (CSCs) were generated in HEK293T packaging cells by co-transfecting the transfer plasmid with the packaging plasmids pVSVg and psPAX2. Viral supernatants were collected 48 h post-transfection, filtered through a 0.45-μm PES membrane, and used to transduce target cells at a 1:1 ratio with fresh medium containing 8 μg/mL polybrene. All plasmids were obtained from MIAOLING BIOLOGY.

The following reagents were purchased from the indicated suppliers: DETA-NONOate, MG132 (proteasome inhibitor), Cycloheximide (protein synthesis inhibitor), MAO-IN-M30 dihydrochloride (MAO inhibitor), L-Methionine-DL-sulfoximine (GLUL inhibitor), BPTES (GLS inhibitor), L-NAME (NOS inhibitor), C75 (FASN inhibitor), and NAC (ROS inhibitor) from MedChemExpress; ARL67156 (CD39 inhibitor), and GW4869 (exosome inhibitor) from Sigma-Aldrich; and JSH23 (NF-κB inhibitor), SCH772984 (ERK inhibitor), and rapamycin (mTOR inhibitor) from TargetMol.

The following assay kits were used: Reactive Oxygen Species Detection Kit (Beyotime, S0033S), Mitochondrial Superoxide Detection Kit (Beyotime, S0061S), Annexin V-FITC/PI Apoptosis Kit (Elabscience, E-CK-A211), and Annexin V-APC/7-AAD Apoptosis Kit (Elabscience, E-CK-A218), Nitric Oxide Assay Kit (Beyotime, S0021S). All reagents and kits were used in accordance with the manufacturers’ instructions.

### Flow cytometry

For intracellular staining, cells were fixed with Fix Buffer I (BD Biosciences) and permeabilized with Perm Buffer III (BD Biosciences). Multiparametric flow cytometry panels were constructed using the following anti-human antibodies: Alexa Fluor^®^ 647 anti-T-bet (BioLegend, 644804), PE anti-GATA3 (BioLegend, 653804), APC anti-RORγt (Invitrogen, 17-6988-82), PE-Cyanine7 anti-FoxP3 (Invitrogen, 25-4776-42), APC/Cyanine7 anti-CD4 (BioLegend, 317450), PE-Cyanine7 anti-CD8 (BioLegend, 344712), APC anti-CD69 (BioLegend, 310910), and CoraLite^®^ Plus 488-Annexin V (Proteintech, PF00005). For quantification of intracellular cytokines, cells were stimulated for 6 hours with PMA (50 ng/mL, Tocris), ionomycin (500 ng/mL, Tocris), and brefeldin A (5 µg/mL, BioLegend), followed by fixation, permeabilization, and staining with APC anti-IL-10 (Biolegend, 501410), PE anti-IFN-γ (BioLegend, 986702), FITC anti-IL-17 (BioLegend, 512306), PE/Cy7 anti-IL-4 (BioLegend, 500810), and FITC anti-Granzyme B (BioLegend, 372206). Staining was performed for 45 minutes at 4 °C in the dark, followed by extensive washing and analysis using flow cytometry with a Canto II instrument (BD Biosciences). Data analysis was conducted using FlowJo software, with fluorescence minus one (FMO) utilized as the gating control.

### Immunoblotting and immunoprecipitation

Cellular proteins were extracted using RIPA buffer (NCM Biotech), and their expression levels were assessed according to standard Western blotting or immunoprecipitation protocols (3, 10, 11). Primary anti-human antibodies were used as follows: Anti-Ki67 antibody (Abcam, ab16667), Anti-Alix (Cell Signaling Technology, 92880), Anti-CD63 (Cell Signaling Technology, 52090), Anti-CD9 (Cell Signaling Technology, 13174), Anti-FoxP3 (Proteintech, 22228-1-AP), Anti-Ubiquitin (Proteintech, 10201-2-AP), and Anti-USP7 (Proteintech, 66514-1-Ig). β-Actin, detected with the corresponding antibody (Santa Cruz Biotechnology, sc-47778), served as an internal control for normalization. A protein molecular weight marker (Epizyme Biomedical Technology, WJ103) was used in these experiments.

### Biotin‐switching assay

The level of S-nitrosylated FoxP3 (SNO-FoxP3) was measured using a biotin switch assay performed with the S-nitrosylation Protein Detection Assay Kit (Cayman, 10006518), following previously described protocols (17, 18). Briefly, cell lysates were first incubated with blocking buffer for 30 minutes to block free thiols, after which proteins were precipitated with cold acetone. The samples were then sequentially treated with reducing buffer and labeling buffer, which converted S-nitrosothiols into free thiols and subsequently labeled them with biotin. Following continuous rotation, biotinylated proteins were captured using streptavidin agarose (Beyotime, P2159) and then subjected to SDS-PAGE followed by immunoblotting with an anti-FoxP3 antibody.

### Real-time PCR

Total RNA was isolated using Trizol (Takara Bio) and then reverse transcribed into cDNA utilizing a reverse transcription Kit (Vazyme Biotech). Quantitative PCR analyses were performed using SYBR Green qPCR Master Mix (Bimake). The primers used are listed in **Table 2**, as previously described, and gene expression was normalized to 18S rRNA (3, 19).

**Table 2:** Sequence of primers targeting different genes for qPCR.

Oligo name	Forward primer (5’ to 3’)	Reverse primer (5’ to 3’)
*18SrRNA*	AGTCCCTGCCCTTTGTACACA	GATCCGAGGGCCTCACTAAAC
*FOXP3*	AGATGGTACAGTCTCTGGAGCAG	AAGTAGTCCATGTTGTGGAGGAA
*NOS2*	GCAGCTCAGCCTGTACT	CACCATCCTCTTTGCGACA
*NOS3*	ACGATGGTGACTTTGGCTA	TGGAGGATGTGGCTGTCT
*NOX1*	CACAAGAAAAATCCTTGGGTCAA	GACAGCAGATTGCGACACACA
*NOX2*	TCACTTCCTCCACCAAAACC	CACCTTCTGTTGAGATCGCC
*NOX3*	CCAGGGCAGTACATCTTGGT	CCGTGTTTCCAGGGAGAGTA
*NOX4*	TGGCTGCCCATCTGGTGAATG	CAGCAGCCCTCCTGAAACATGC
*NOX5*	CAGGCACCAGAAAAGAAAGCAT	ATGTTGTCTTGGACACCTTCGA
*GLS1*	GTCACGATCTTGTTTCTCTGTG	GTCCAAAGAGCAGTGCTTCATCCATG
*GLS2*	TGCCTATAGTGGCGATGTCTCA	GTTCCATATCCATGGCTGACAA
*GLUL*	CCTGCTTGTATGCTGGAGTC	GATCTCCCATGCTGATTCCT
*USP7*	CGTTCGGAATCCCGTTTTTGCT	TCAAGGTAAGTGTAGCGACTCC
*USP11*	GAAGAGAACGGACGGCGAT	CGTGCTGTGGCTCTCTATCC
*USP21*	AGGTGTCTCTGCGGGATTGTT	CGATTCAGATGGAGCACGAGG
*USP44*	ACAACTTATGATATGCCACC	GATTTCCTCAAAGCCAAC
*USP19*	CGGCACAAGATGAGGAATGA	GGCACCGGCAGATAAAGAAA
*USP10*	TTTTAAATGCCACCGAACCTATC	CCAGCCATTCAGACCGATCT
*USP3*	GTTTCAACGGTGTTTCCC	AATGCCTCCGAATATAGCC
*USP33*	AAAATCCCTTGGTACTTGTCAGG	TCGAAGAGTGGTAAGGTTCACA
*USP9X*	CAATGGATAGATCGCTTTATA	CTTCTTGCCATGGCCTTAAAT
*USP17L2*	ACACTTTTGACCCTTACCTGG	GCTTCACCAACTGTTCCAAAG

### Exosome and macrovesicle isolation

Cell culture supernatants were collected and subjected to sequential centrifugation to remove cells and debris: first at 200 × g for 10 min, then at 2,700 × g for 10 min, and finally at 14,000 × g for 2 min. The resulting supernatant was then divided for parallel isolation of macrovesicles and exosomes. For macrovesicle isolation, the supernatant was centrifuged at 14,000 × g for 1 h, and the pellet was collected as the macrovesicle fraction.

For exosome isolation, the supernatant was filtered through 0.22-μm filters and ultracentrifuged at 100,000 × g (or 31,000 rpm) for 70 min at 4 °C. The resulting exosome pellets from multiple tubes were pooled, resuspended in PBS, and subjected to a second ultracentrifugation under the same conditions. The final exosome pellet was resuspended in PBS and quantified using the ExoELISA-ULTRA Complete Kit (System Biosciences). Exosome identity was further confirmed by immunoblotting for the exosomal markers Alix, CD63, and CD9 (20).

### Patient-derived organoids (PDOs)

PDOs were derived from NSCLC tissue specimens obtained from resected patients, following previously established protocols (3, 10, 11). Specifically, NSCLC tissue samples were minced into a paste-like consistency, treated with red blood cell lysis buffer, suspended in PDOs medium, and placed in ultralow adsorption culture plates (Corning). The plates were then placed on an orbital shaker (120 rpm) and incubated in a 37 °C incubator with 5% CO2. The culture medium was replenished twice weekly. Verification of PDOs was performed using anti-human PanCK (Abcam, ab7753), anti-human CD31 (Abcam, 281583) antibodies, and histological analyses.

### The PDO-PBMC co-culture

Patient-derived organoids (PDOs) were randomly allocated into control and treatment groups. Three experimental conditions were set up: (1) untreated PDOs were co-cultured with CD4^+^ T cells; (2) PDOs were pretreated with the GLUL inhibitor MSX and then co-cultured with autologous CD4^+^ T cells; (3) CD4^+^ T cells co-cultured with untreated PDOs were subsequently treated with the NOS inhibitor L-NAME. After co-culture, CD4^+^ T cells were separated from PDOs, reconstituted with the remaining autologous PBMC components, and the function of CD8^+^ T cells within the reconstituted PBMCs was assessed. To monitor PDO growth, PBMCs from NSCLC patients were co-cultured with control or BPTES-treated PDOs, and organoid growth was monitored under bright-field microscopy.

### Immunofluorescence

Lung tissues and PDOs from NSCLC patients were embedded, sectioned, and fixed with acetone. Subsequently, the sections were blocked with BSA and immunostained with anti-CD31 antibody (abcam, ab28364) and anti-pan cytokeratin (Pan CK) antibody (abcam, ab215838). Nuclei were counterstained with DAPI (beyotime, C1002).

### Statistics

The data were expressed as mean ± SEM. Paired and unpaired t tests were employed for comparisons between two groups. One-way ANOVA with the Tukey method was utilized for comparisons involving more than two groups. The exact number of times experiment repeated for each panel was indicated in figure legends. Statistical analyses were performed using PRISM 9.0 (GraphPad Software Inc.), with significance defined as *p* < 0.05.

## Results

### CSCs promote the differentiation of Treg cells while inhibiting effector T cells

To investigate the effect of CSCs on CD4^+^ T cell differentiation, we first enriched CSCs via sphere culture (**Supplementary Figure S1A**). The CSC identity was verified by elevated expression of stemness markers including CD133, SOX2, OCT4, ALDH1A (**Supplementary Figure S1B**), increased CD133^+^ cell frequency, enhanced ALDH activity, and stronger tumorigenic capacity (**Supplementary Figures S1C–E**).

CSCs were then co-cultured with T cells from healthy donors for 12 hours, followed by T cell activation with anti-CD3/CD28 beads. Notably, CSC pre-incubation reduced the frequencies of T-bet^+^ Th1, GATA3^+^ Th2, and RORγt^+^ Th17 cells, while significantly promoting FoxP3^+^ Treg cell generation (**Figure 1A**). Importantly, CSCs did not affect T cell activation, as indicated by unchanged CD69 expression (**Supplementary Figure S2A**). The observed increase in Treg frequency was not attributable to enhanced survival or proliferation, because both apoptosis and proliferation remained unaltered in total CD4^+^ T cells and CD4^+^FoxP3^+^ T cells under CSC-primed conditions (**Supplementary Figures S2B–D**). To validate clinical relevance, CD133^+^ CSCs isolated from cultured patient-derived organoids (PDOs) (**Supplementary Figures S3A–C**) were co-cultured with CD4^+^ T cells and stimulated with anti-CD3/CD28 beads; consistent with previous results, CSCs enhanced FoxP3^+^ Treg differentiation (**Figure 1B**).

**Figure 1:**
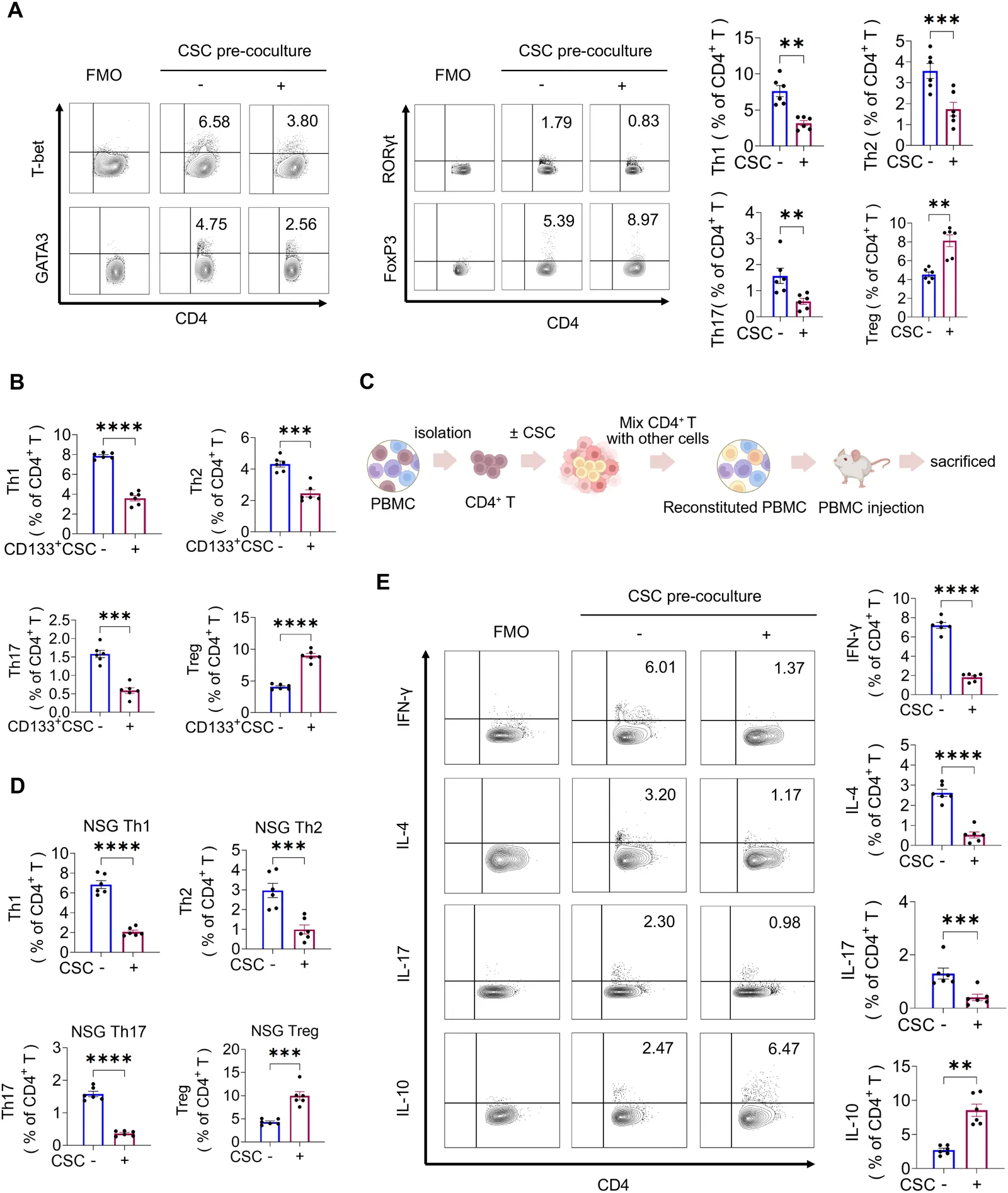
CSCs drive the differentiation of Treg cells. **(A)** Healthy CD4^+^ T cells were pre-conditioned with or without CSCs for 12 hours, activated with anti-CD3/CD28 beads for 4 days, and tested for cell subsets by quantifying the indicated T-bet^+^ Th1, GATA3^+^ Th2, RORγt^+^ Th17 and FoxP3^+^ Treg cells using flow cytometry. Mean ± SEM from 6 individuals in each group. **(B)** Healthy CD4^+^ T cells from healthy individuals were pre-conditioned with or without PDO-derived CD133^+^ CSCs for 12 hours, followed by activation with anti-CD3/CD28 beads for 4 days. Mean ± SEM from 6 individuals in each group. **(C)** Schematic diagram of the humanized T cell-reconstituted NSG mouse model. **(D, E)** Healthy CD4^+^ T cells from PBMCs were pre-conditioned with or without CSCs for 12 hours, reintroduced into PBMCs, followed by adoptive transferred into immune-deficient NSG mice (10 million cells per mouse). The splenic frequencies of the indicated T cell subsets and associated cytokines were analyzed after 7 days by flow cytometry. Mean ± SEM from 6 chimeras in each group. **p < 0.01, ***p < 0.001, and ****p < 0.0001 with paired t-tests **(A–B, D–E)**.

To further confirm the role of CSCs in regulating T cell differentiations *in vivo*, CD4^+^ T cells isolated from healthy donor peripheral blood mononuclear cells (PBMCs) were pre-cocultured with or without CSCs, reintroduced into the PBMC population, and adoptively transferred into immunodeficient NSG mice (**Figure 1C**). Mice receiving CSC-pretreated T cells exhibited higher frequencies of splenic Treg cells and their associated cytokines (**Figures 1D, E**), whereas splenic effector T cells and their associated cytokines were reduced (**Figures 1D, E**). Collectively, CSCs efficiently regulate T cell differentiation, promoting Treg generation and establishing an inhibitory immune microenvironment in the TME.

### CSCs program T cell differentiation in an exosome-dependent manner

To investigate whether CSCs regulate T cell differentiation in a cell-cell contact-dependent manner, we performed a Transwell co-culture assay. Consistent with previous observations, CSCs effectively promoted FoxP3^+^ Treg cell differentiation while impairing the differentiation of T-bet^+^ Th1 and RORγt^+^ Th17 cells (**Figure 2A**), indicating that CSC-mediated T cell differentiation modulation is independent of direct cell-cell contact.

**Figure 2:**
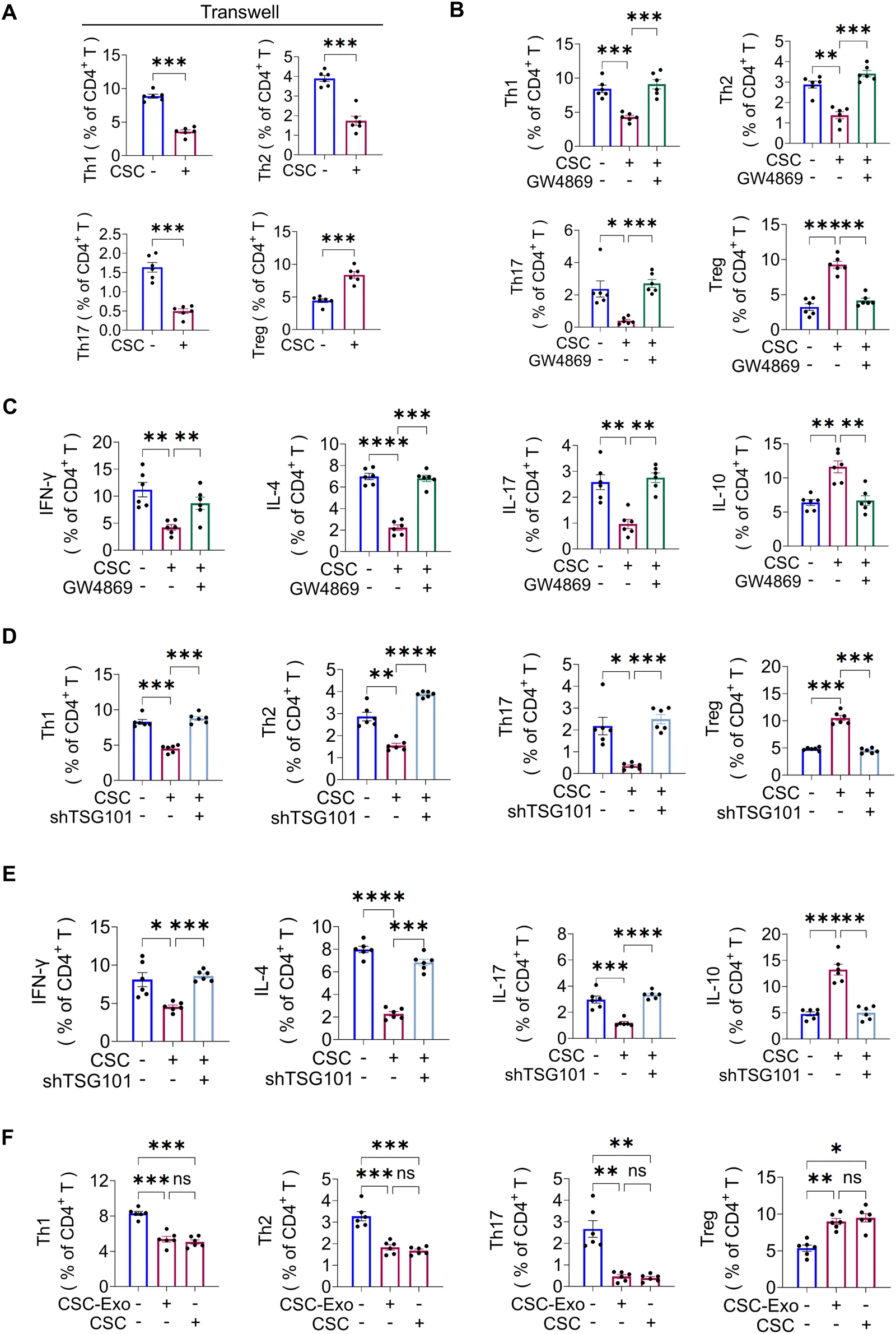
The effect of CSCs on T cells is independent of cell-cell contact. **(A)** CSCs were co-cultured with healthy CD4^+^ T cells using a Transwell assay for 12 hours, followed by T cell activation for 4 days. Mean ± SEM from 6 individuals. **(B, C)** CSCs were pretreated with or without the exosome inhibitor GW4869 (10 μM) for 24 hours, followed by co-culture with T cells and subsequent T cell activation for 4 days. Mean ± SEM from 6 individuals. **(D, E)** CSCs were transfected with TSG101 shRNA for 24 hours, followed by co-culture with T cells and T cell activation for 4–6 days. Mean ± SEM from 6 independent experiments. **(F)** Healthy CD4^+^ T cells were stimulated with anti-CD3/CD28 beads in the presence of CSC-derived exosomes or co-cultured with CSCs. Mean ± SEM from 6 independent experiments. *p < 0.05, **p < 0.01, ***p < 0.001, ****p < 0.0001 with paired **(A)** and ANOVA with Tukey’s method **(B–F)**.

Pretreatment of CSCs with GW4869, an exosome secretion inhibitor, abrogated their ability to regulate T cell differentiation and the secretion of associated cytokines (**Figures 2B, C**). We next knocked down TSG101, a gene involved in vesicle formation (**Supplementary Figure S4A**), which also eliminated the effect of CSCs on Treg cell differentiation (**Figures 2D, E**). Notably, exosomes isolated from CSC-conditioned medium alone were sufficient to drive aberrant differentiation of Th cell subsets (**Figure 2F**; **Supplementary Figure S4B**), whereas isolated macrovesicles showed no such effects (**Supplementary Figures S4C, D**). This finding was further validated by the observation that exosome-depleted CSC supernatant failed to affect the differentiation of FoxP3^+^ Treg (**Supplementary Figure S4E**). Collectively, these results demonstrate that CSCs instruct T cell differentiation in an exosome-dependent manner.

### Elevated glutamine metabolism in CSCs generates excessive ROS to drive Treg cell differentiation

Given that IL-10 and TGF-β are well-established inducers of tumor immune escape (21), we sought to determine whether CSC-induced Treg differentiation is mediated by these cytokine pathways. To this end, we separately knocked down TGFB1 or IL-10 in CSCs and then co-cultured them with T cells. Neither knockdown reversed the CSC-mediated enhancement of Treg differentiation (**Supplementary Figure S5A**), indicating that the Treg-promoting effect of CSCs is largely independent of IL-10 and TGF-β signaling. Previous studies have shown that NSCLC non-CSCs induce Treg cells through a CD39-dependent mechanism (3). We therefore tested whether CD39 inhibition in CSCs prior to T cell co-culture alters their regulatory function in T cell differentiation. Interestingly, neither shRNA-mediated knockdown nor pharmacological inhibition of CD39 in CSCs significantly affected the subsequent differentiation of FoxP3^+^ Tregs (**Supplementary Figures S5B–D**), although the precise mechanisms remain to be fully elucidated.

We next performed RNA sequencing on NSCLC CSCs and non-CSCs (National Genomics Data Center, HRA001456), which revealed distinct pathway enrichments in CSCs, including ROS-related pathways (**Supplementary Figure S6**). Consistent with this, the ROS pathway was significantly upregulated in CSCs (**Figure 3A**), and CSCs exhibited higher ROS levels than non-CSCs (**Figure 3B**). To assess the functional relevance of CSC-derived ROS, we treated T cells with H2O2. H2O2 treatment suppressed the differentiation of T-bet^+^ Th1, GATA3^+^ Th2, and RORγt^+^ Th17 cells, while promoting FoxP3^+^ Treg differentiation (**Supplementary Figure S7A**). Importantly, pretreatment of CSCs with the ROS scavenger N-acetylcysteine (NAC) abrogated their ability to promote FoxP3^+^ Treg generation (**Figure 3C**; **Supplementary Figure S7B**) and reversed the CSC-induced suppression of Th1, Th2, and Th17 cells (**Figure 3C**), establishing a key role for CSC-derived ROS in modulating T cell differentiation. We further confirmed that primary CSCs isolated from NSCLC patients also exhibited higher intracellular ROS levels than their non-CSC counterparts (**Figure 3D**). Similarly, NAC pretreatment of primary CSCs abolished their effects on the differentiation of FoxP3^+^ Tregs and T-bet^+^ Th1 cells (**Figure 3E**). Collectively, these findings demonstrate that CSCs are enriched in ROS and utilize this mechanism to regulate T cell differentiation.

**Figure 3:**
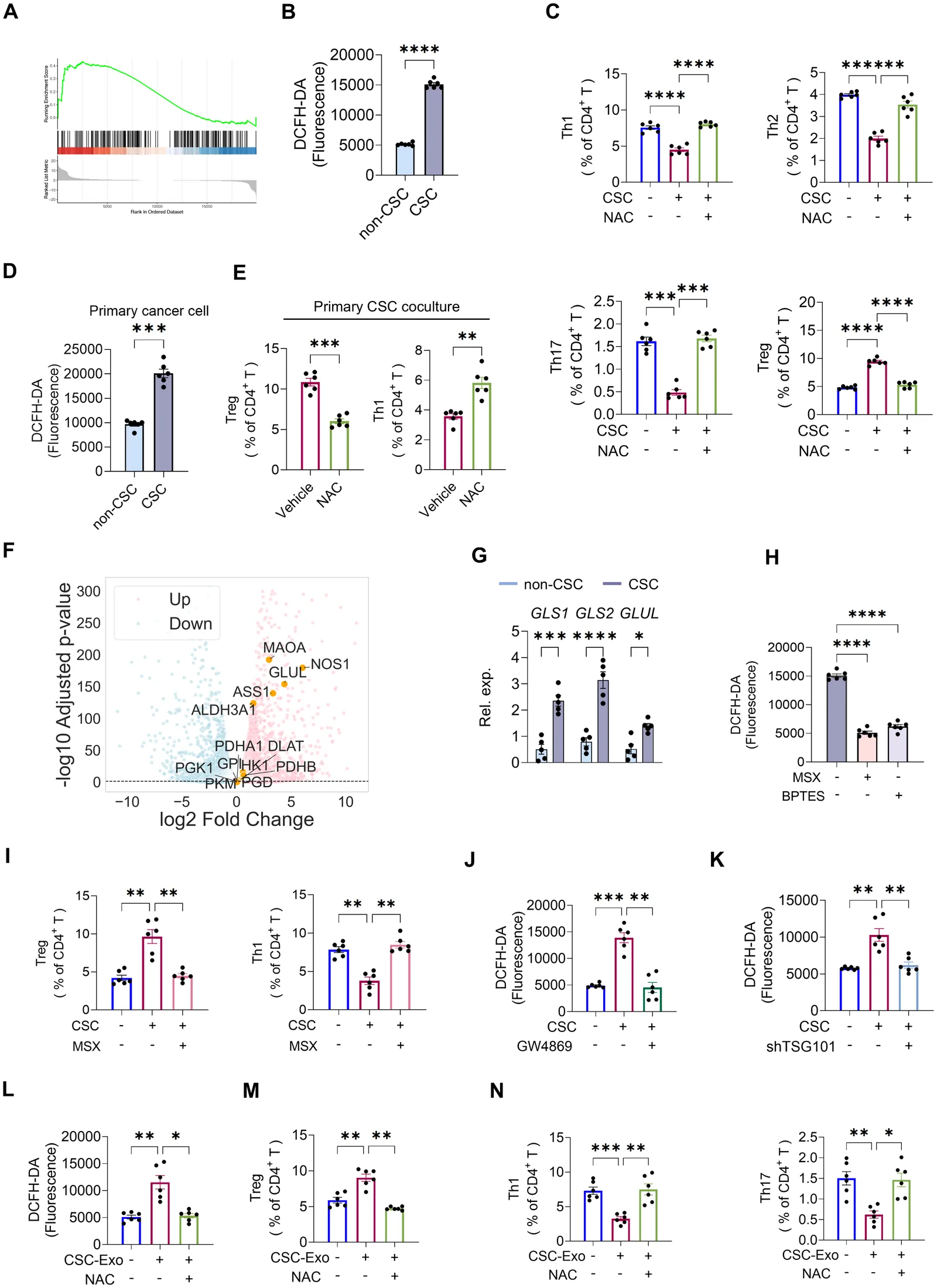
High ROS resulting from active glutamine metabolism in CSCs regulates T cell differentiation. **(A)** RNA-sequencing analysis revealed upregulation of ROS-related pathways in CSCs. **(B)** Intracellular ROS levels in CSCs and non-CSCs isolated from the A549 cell line were detected using DCFH-DA. Mean ± SEM from 6 independent experiments. **(C)** CSCs were pretreated with or without ROS inhibitor NAC (10 μM) for 24 hours, followed by 12-hour CSC-T cell co-culture and T cell activation for 4 days. Mean ± SEM from 6 independent experiments. **(D)** Intracellular ROS levels in CD133^+^ CSCs and CD133^-^ non-CSCs from NSCLC patients were detected using DCFH-DA. Mean ± SEM from 6 individuals. **(E)** Primary CD133^+^ CSCs were pretreated with or without ROS inhibitor NAC (10 μM) for 24 hours, followed by 12-hour CSC-T cell co-culture and T cell activation for 4 days. Mean ± SEM from 6 independent experiments. **(F)** The volcano plot presented the analysis results from bulk RNA sequencing, illustrating elevated expression of genes involved in glutamine metabolism in CSCs. Each point represents a gene. **(G)** mRNA expression levels of GLS1, GLS2, and GLUL in non-CSCs and CSCs. Mean ± SEM from 5 independent experiments. **(H)** CSCs were pretreated with or without MSX (1 mM) or BPTES (1 μM) for 24 hours, and intracellular ROS levels were detected. Mean ± SEM from 6 independent experiments. **(I)** CSCs were pretreated with or without MSX (1 mM) for 24 hours, followed by 12-hour CSC-T cell co-culture and T cell activation for 4 days. Mean ± SEM from 6 independent experiments. **(J)** CSCs were pretreated with or without exosome inhibitor GW4869 (10 μM) for 24 hours, followed by CSC-T cell co-culture for 12 hours. Intracellular ROS levels were detected from 6 individuals. **(K)** CSCs were transfected with TSG101 shRNA for 24 hours, followed by CSC-T cell co-culture for 12 hours. Intracellular ROS levels were detected from 6 individuals. **(L)** Exosomes isolated from CSCs +/- NAC pretreatment were added to the T cells for 12 hours. Intracellular ROS levels were detected from 6 individuals. **(M, N)** Exosomes isolated from CSCs +/- NAC pretreatment were added to the T cell activation culture for 4 days. Mean ± SEM from 6 individuals. *p < 0.05, **p < 0.01, ***p < 0.001, ****p < 0.0001 with paired **(E)** and unpaired **(B, D)** t-tests or ANOVA with Tukey’s method **(C, G–N)**.

To explore the mechanisms underlying elevated ROS levels in CSCs, we examined key genes involved in ROS generation, including NADPH oxidases (NOX) and mitochondrial ROS (22, 23). The expression of NOX isoforms (NOX1-5) was comparable between CSCs and non-CSCs (**Supplementary Figure S7C**), suggesting that the NOX family is not a major contributor to the increased ROS levels in CSCs. Notably, mitochondrial ROS levels were significantly elevated in CSCs (**Supplementary Figure S7D**), indicating that mitochondrial ROS may be the primary driver mediating the observed effects on Treg cell differentiation.

CSCs exhibit distinct metabolic profiles from non-CSCs, with heightened lipogenesis observed in human NSCLC (10). However, inhibition of lipogenesis using C75, a fatty acid synthase (FAS) inhibitor, did not significantly impact mitochondrial ROS levels in CSCs (**Supplementary Figure S7E**). RNA-seq analysis revealed upregulated GLUL expression in CSCs (**Figure 3F**), and qPCR results confirmed that enzymes involved in both glutamine synthesis and catabolism are enriched in CSCs (**Figure 3G**). Blocking glutamine metabolism with either the glutamine synthetase (GLUL) inhibitor L-Methionine-DL-sulfoximine (MSX) or the glutaminase 1 (GLS1) inhibitor BPTES effectively attenuated mitochondrial ROS generation in CSCs (**Supplementary Figure S7F**), resulting in reduced intracellular ROS levels (**Figure 3H**). Additionally, pretreatment with MSX impaired the ability of CSCs to regulate the differentiation of Treg and Th1 cells (**Figure 3I**). Of note, RNA-seq data also revealed that MAOA, a mitochondrial enzyme that produces H2O2 during monoamine catabolism (24), was upregulated in CSCs. To dissect the functional contribution of MAOA-derived ROS to CSC-mediated Treg induction, we pharmacologically inhibited MAOA activity in CSCs. However, MAOA inhibition only moderately decreased mitochondrial ROS accumulation in CSCs and failed to abrogate CSC-induced Treg enhancement or rescue impaired Th1 differentiation (**Supplementary Figure S7G-H**), suggesting that MAOA is not a major contributor to the observed CSC effects.

To further validate the role of CSC-derived exosomal ROS in T cell differentiation, we pretreated CSCs with GW4869 or shRNA targeting TSG101. The results showed that CSCs transferred ROS to T cells, and this effect was abrogated by exosome inhibition (**Figures 3J, K**). Isolation of exosomes from CSCs demonstrated that these CSC-derived exosomes increased ROS levels in T cells; this effect was reversed by NAC (**Figure 3L**), which in turn restored the abnormal differentiation of T cells (**Figures 3M, N**). Collectively, these findings confirm that CSC-derived exosome-encapsulated ROS promote FoxP3^+^ Treg cell differentiation.

### ROS efficiently induces FoxP3 deubiquitination to guide T cell differentiation

To explore the mechanism by which CSC-transferred ROS induces T cell mal-differentiation, we next detected FoxP3 expression levels in T cells pretreated with CSCs or H2O2. Our results showed that both CSC and H2O2 treatment significantly enhanced FoxP3 protein expression (**Figures 4A, B**), whereas mRNA levels remained unchanged (**Supplementary Figure S8A**). A cycloheximide (CHX) chase assay revealed markedly retarded FoxP3 degradation in CSC-exposed T cells compared to controls (**Supplementary Figure S8B**), providing direct kinetic evidence that CSC exposure enhances FoxP3 protein stability. MG132 treatment confirmed that FoxP3 degradation occurs via the ubiquitin–proteasome pathway (**Figure 4C**). Notably, pretreatment of CSCs with MSX reversed the CSC-induced elevation of FoxP3 expression (**Figure 4D**). We further examined FoxP3 ubiquitination in T cells following CSC or H2O2 treatment. The results demonstrated that CSC or H2O2 treatment significantly reduced FoxP3 ubiquitination (**Figures 4E, F**), whereas CSCs pretreated with MSX lost the ability to decrease FoxP3 ubiquitination (**Figure 4G**).

**Figure 4:**
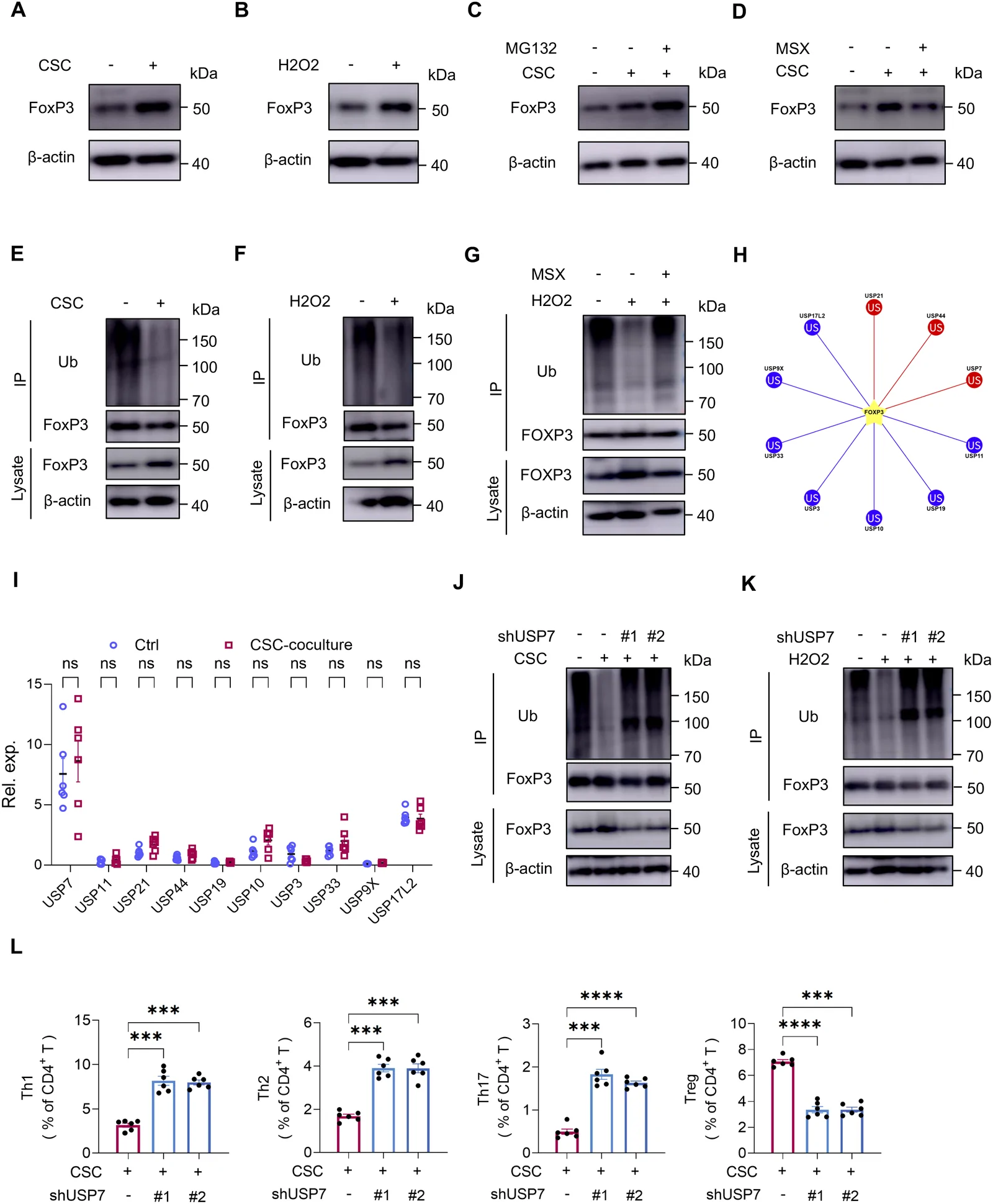
ROS instruct FoxP3 deubiquitination in T cells. **(A)** Healthy CD4^+^ T cells were co-cultured with CSCs for 12 hours, followed by stimulation with anti-CD3/CD28 beads for 4 days. FoxP3 protein expression was quantified by western blot. **(B)** Healthy CD4^+^ T cells were stimulated with anti-CD3/CD28 beads for 4 days in the presence or absence of H2O2 (10 μM). FoxP3 protein expression was quantified by western blot. **(C)** Healthy CD4^+^ T cells were co-cultured with CSCs for 12 hours, then stimulated with anti-CD3/CD28 beads for 4 days. During induction, cells were treated with the proteasome inhibitor MG132 (2 μM) or vehicle control for 4 hours. FoxP3 protein expression was quantified by western blot. **(D)** CD4^+^ T cells were co-cultured with CSCs pretreated with or without MSX for 24 hours, followed by stimulation with anti-CD3/CD28 beads for 4 days. FoxP3 protein expression was quantified by western blot. **(E)** Endogenous FoxP3 was immunoprecipitated from CD4^+^ T cells with or without CSC priming to assess its ubiquitination profile. **(F)** Endogenous FoxP3 was immunoprecipitated from CD4^+^ T cells following H2O2 (10 μM) stimulation to examine its ubiquitination levels. **(G)** CD4^+^ T cells were co-cultured with CSCs pretreated with or without MSX for 24 hours, followed by stimulation with anti-CD3/CD28 beads for 4 days. Endogenous FoxP3 was immunoprecipitated from CD4^+^ T cells to analyze its ubiquitination. **(H)** Potential DUBs of FoxP3 were predicted using the UbiBrowser database. **(I)** qPCR was performed to detect the mRNA levels of the DUBs in CD4^+^ T cells activated for 4 days. Mean ± SEM from 6 individuals in each group. **(J)** CD4^+^ T cells were transfected with USP7 shRNA for knockdown or control shRNA, then co-cultured with CSCs for 12 hours, followed by stimulation with anti-CD3/CD28 beads for 4 days. FoxP3 protein expression was quantified by western blot. **(K)** CD4^+^ T cells were transfected with USP7 shRNA for knockdown or control shRNA, then stimulated with anti-CD3/CD28 beads in the presence or absence of H2O2 (10 μM) for 4 days. FoxP3 protein expression was quantified by western blot. **(L)** CD4^+^ T cells were transfected with USP7 shRNA for knockdown or control shRNA, then co-cultured with CSCs for 12 hours, followed by stimulation with anti-CD3/CD28 beads for 4 days. The proportion of T cell differentiation was analyzed by FCS. Mean ± SEM from 6 independent experiments. Mean ± SEM from 6 independent experiments. ***p < 0.001 and ****p < 0.0001 with ANOVA with Tukey’s method **(L)**.

We next employed Ubibroser to predict deubiquitinating enzymes (DUBs) of FoxP3 (**Figure 4H**), and our results showed that only USP7 was highly expressed in CD4^+^ T cells (**Figure 4I**). Knockdown of USP7 via shRNA restored FoxP3 ubiquitination in T cells treated with CSCs or H2O2 (**Figures 4J, K**) and, importantly, reversed T cell mal-differentiation (**Figure 4L**; **Supplementary Figure S9**). Collectively, these findings pinpoint a crucial role of CSC-derived exosomal ROS in sustaining FoxP3 expression levels to promote Treg cell differentiation.

### ROS upregulates NO levels in T cells to mediate FoxP3 S-nitrosylation and deubiquitination

To investigate how ROS regulates FoxP3 ubiquitination in T cells, we treated T cells with inhibitors of ROS-associated downstream signaling pathways involved in T cell differentiation (25–27), including rapamycin (mTOR inhibitor), SCH772984 (ERK inhibitor), JSH23 (NF-κB inhibitor), and L-NAME (NO synthase inhibitor). Among these, only L-NAME effectively blocked H2O2-induced upregulation of FoxP3 protein levels (**Supplementary Figure S10A**). Subsequent experiments showed that both CSC and H2O2 treatment significantly increased NO levels in T cells (**Figures 5A, B**), an effect reversed by scavenging ROS from CSCs (**Figure 5C**). The observed NO increase was attributable to enhanced iNOS expression, rather than eNOS (**Supplementary Figure S10B**). Notably, inhibiting NO synthesis in CSCs did not affect T cell NO levels (**Figure 5D**), whereas direct L-NAME treatment of T cells did (**Figure 5E**), suggesting that CSC-mediated NO upregulation in T cells is not due to direct NO transfer from CSCs.

**Figure 5:**
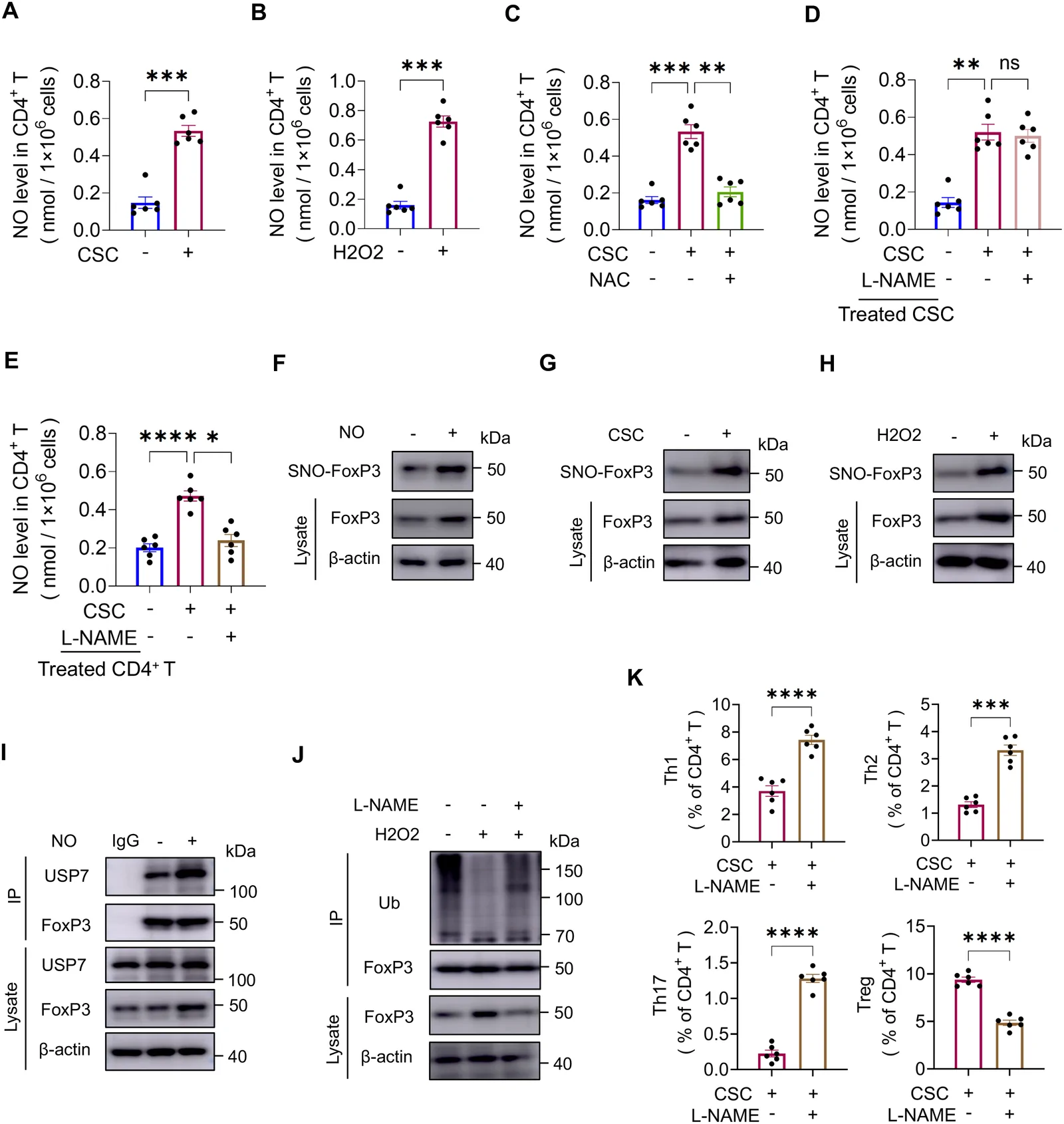
ROS enhance NO levels in CD4^+^ T cells to induce FoxP3 S-nitrosylation and deubiquitination. **(A)** Healthy CD4^+^ T cells were co-cultured with CSCs for 12 hours, followed by stimulation with anti-CD3/CD28 beads for 4 days. NO levels were quantified using an assay kit. **(B)** Healthy CD4^+^ T cells were stimulated with anti-CD3/CD28 beads for 4 days in the presence or absence of H2O2 (10 μM). NO levels were quantified using an assay kit. **(C)** CD4^+^ T cells were co-cultured with CSCs pretreated with or without NAC (10 μM) for 24 hours, followed by stimulation with anti-CD3/CD28 beads for 4 days. NO levels were quantified using an assay kit. **(D)** CD4^+^ T cells were co-cultured with CSCs pretreated with or without L-NAME (100 μM) for 24 hours, followed by stimulation with anti-CD3/CD28 beads for 4 days. NO levels were quantified using an assay kit. **(E)** CD4^+^ T cells were co-cultured with CSCs for 12 hours, followed by stimulation with anti-CD3/CD28 beads for 4 days in the presence or absence of L-NAME (100 μM). NO levels were quantified using an assay kit. **(F)** SNO-FoxP3 levels in CD4^+^ T cells treated with DETA NONOate (20 μM, 4 days) were detected by biotin switch assay. **(G)** SNO-FoxP3 levels in CD4^+^ T cells treated with CSCs were detected by biotin switch assay. **(H)** SNO-FoxP3 levels in CD4^+^ T cells treated with H2O2 (10 μM, 4 days) were detected by biotin switch assay. **(I)** Co-immunoprecipitation analysis was performed to examine the interaction between FoxP3 and USP7 in CD4^+^ T cells treated with DETA NONOate (20 μM, 4 days). **(J)** Immunoprecipitation analysis was performed to detect FoxP3 ubiquitination in CD4^+^ T cells treated with H2O2 (10 μM) with or without L-NAME (100 μM). **(K)** CD4^+^ T cells were co-cultured with CSCs for 12 hours, followed by stimulation with anti-CD3/CD28 beads for 4 days in the presence or absence of L-NAME (100 μM). The proportion of T cell differentiation was analyzed by FCS. *p < 0.05, **p < 0.01, ***p < 0.001, ****p < 0.0001 with paired **(A, B, K)** t-tests or ANOVA with Tukey’s method **(C–E)**.

Given that NO often modulates protein expression and function via S-nitrosylation (**Figure 5F**), we examined FoxP3 S-nitrosylation following CSC or H2O2 treatment. Both conditions markedly induced FoxP3 nitrosylation (**Figures 5G, H**). To explore the link between FoxP3 S-nitrosylation and deubiquitination, we assessed FoxP3-USP7 interaction after NO treatment. NO significantly enhanced the association between FoxP3 and USP7 (**Figure 5I**). Furthermore, L-NAME treatment reversed the reduction in FoxP3 ubiquitination in H2O2-pretreated T cells (**Figure 5J**). Similarly, L-NAME treatment restored the CSC-induced aberrant differentiation of Th subsets in T cells pre-educated by CSCs (**Figure 5K**). Collectively, these results indicate that CSC-derived ROS upregulates NO levels in T cells, which in turn promotes FoxP3 S-nitrosylation and deubiquitination, ultimately driving Treg cell differentiation.

### Targeting the glutamine metabolism-ROS-FOXP3 axis restores CD8^+^ T cell function and suppresses tumor growth in human NSCLC

To evaluate the functional and clinical relevance of the glutamine metabolism-ROS-FOXP3 axis in tumor progression, we established a series of co-culture assays using patient-derived tumor organoids (PDOs) and autologous CD4^+^ T cells. CD4^+^ T cells were first co-cultured with PDOs either untreated or pretreated with MSX. In parallel, CD4^+^ T cells exposed to untreated PDOs were subsequently treated with L-NAME. These manipulated CD4^+^ T cells were then reconstituted with the remaining autologous PBMC components, and the resulting PBMC populations were co-cultured with conventional tumor cells to assess their cytotoxic activity (**Figure 6A**). We found that MSX pretreatment of PDOs reversed the PDO-induced reduction in PBMC cytotoxicity (**Figures 6B, C**). Similarly, L-NAME treatment of CD4^+^ T cells enhanced the tumor-killing capacity of PBMCs (**Figures 6B, C**). These findings support a critical role of the glutamine metabolism-ROS-FOXP3 axis in suppressing anti-tumor immunity.

**Figure 6:**
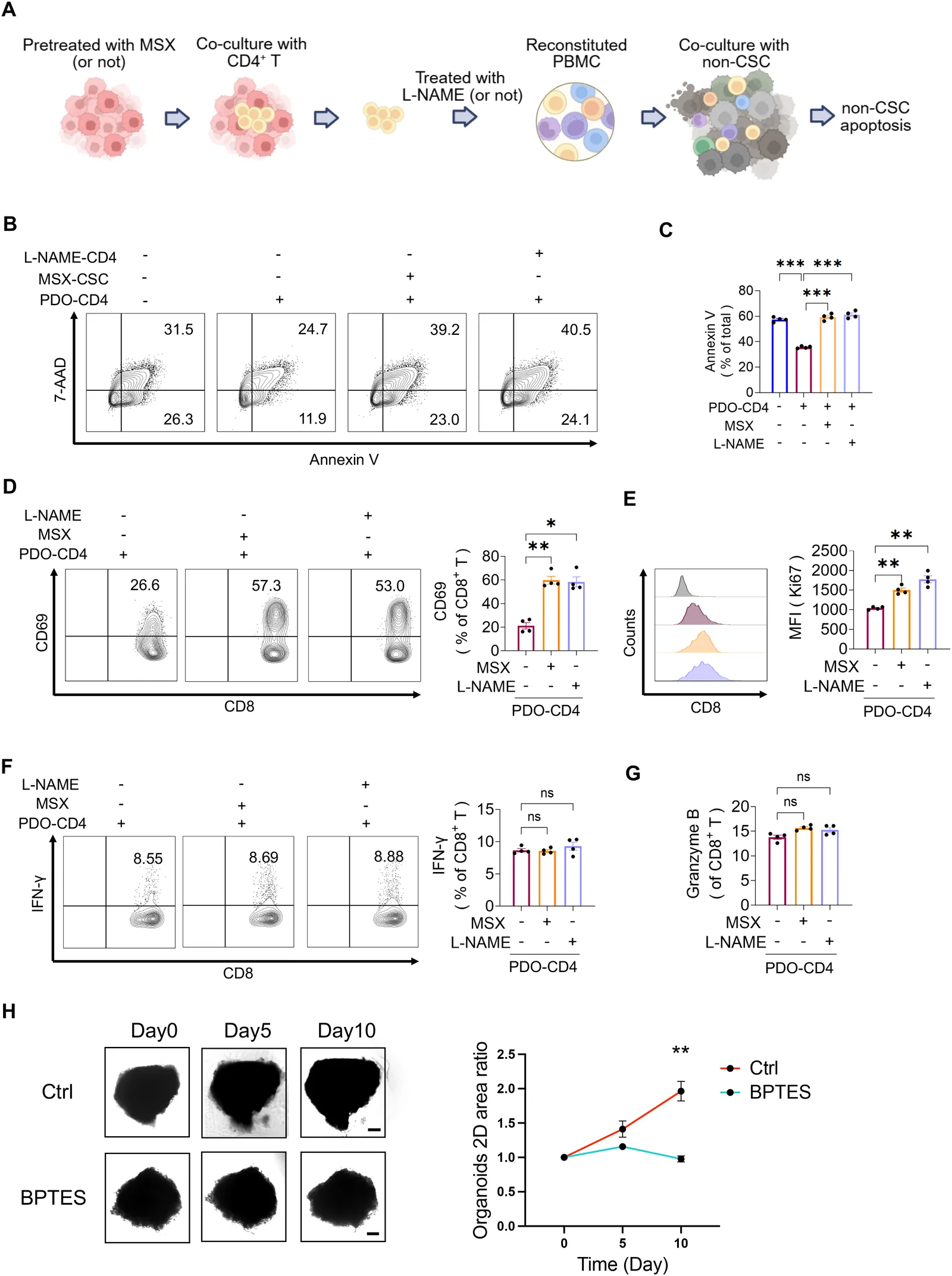
Targeting glutamine metabolism restrains CD8^+^ T cell function and restricts tumor growth in PDOs. **(A)** Schematic diagram of the effect of CD4^+^ T cells in the TME on CD8^+^ T cell killing function. **(B-C)** Percentage of Annexin V^+^ A549 cells following incubation with reconstituted PBMCs containing PDO-conditioned CD4^+^ T cells or control PBMCs for 96 hours. Mean ± SEM from 4 individuals. **(D–G)** CD4^+^ T cells were co-cultured with PDOs pretreated with or without MSX, then treated with L-NAME, and subsequently reconstituted into PBMCs. CD8^+^ T cell activation (CD69^+^) was analyzed after 24 hours **(D)**, CD8+ T cell proliferation (Ki67^+^) was detected after 3 days **(E)**, and cytokine secretion (IFN-γ^+^ or granzyme B^+^) was detected after 4 days **(F, G)**. Mean ± SEM from 4 independent experiments. **(H)** PDOs were treated with or without BPTES (10 μM) for 24 hours, followed by co-culture with PBMCs for 10 days. PDO tumor growth was measured at the indicated time points. Mean ± SEM from 4 independent experiments. *p < 0.05, **p < 0.01 and ***p < 0.001 with ANOVA with Tukey’s method **(C–H)**.

We next assessed the activation, proliferation, and function of CD8^+^ T cells within the reconstituted PBMC population. Both MSX-pretreated organoids and L-NAME-treated CD4^+^ T cells restored the activation and proliferation of CD8^+^ T cells (**Figures 6D, E**) but did not affect their cytokine production (**Figures 6F, G**). Furthermore, treatment with GLS inhibitor BPTES suppressed organoid growth (**Figure 6H**). In summary, targeting glutamine metabolism proves to be an effective strategy in rectifying the aberrant differentiation of T cells and restraining the growth of human NSCLC.

## Discussion

Current research regarding CSCs predominantly focuses on understanding their roles in tumorigenesis, tumor progression, treatment resistance, and metastasis, while the potential impact of CSCs on anti-tumor immunity remains largely unexplored (13, 28). In this study, we uncover a novel function of CSCs in promoting Treg cells in human NSCLC, a function shared with non-CSCs. Nevertheless, the mechanisms by which CSCs and non-CSCs regulate T cell differentiation differ. Non-CSCs transfer CD39 protein to T cells, decreasing ATP levels and activating AMPK to induce Treg cells (3). Conversely, CSCs utilize exosomal ROS to promote Treg cell differentiation. Mechanistically, CSCs exhibit heightened gluatamine metabolism, leading to increased mitochondrial ROS to regulate the T cell differentiations.

The role of ROS in CSCs is intricate and context-dependent, exerting diverse regulatory effects on CSC biological behaviors and tumor progression (29). In breast CSCs, sustained low ROS levels—driven by nuclear factor erythroid-derived 2-like 2 (Nrf2) overactivation—promote the upregulation and accumulation of nuclear FoxO3a, as well as its subsequent binding to the Bmi-1 promoter. This regulatory cascade effectively preserves the self-renewal capacity of BCSCs and promotes their xenograft growth *in vivo*, providing direct evidence that low ROS environments are conducive to the maintenance of CSC stemness (30). Conversely, high ROS levels can also serve as a driver of CSC stemness, accompanied by unique antioxidant adaptations that enable CSCs to survive and function in an oxidative microenvironment. In triple-negative breast cancer (TNBC), oncogenes MYC and MCL1 enhance mitochondrial oxidative phosphorylation, leading to elevated ROS production; this high-ROS state further promotes CSC stemness and chemoresistance, and targeted inhibition of this pathway can effectively reverse chemotherapy resistance (31). Similarly, glioblastoma CSCs inherently exhibit high intracellular ROS levels and preferentially express NAD(P)H quinone dehydrogenase 1 (NQO1)—a key enzyme not only essential for GSC proliferation and self-renewal but also critical for conferring resistance to oxidative stress. This unique antioxidant mechanism allows GSCs to maintain their stemness and survive stably in a high-ROS milieu (32). Consistent with the well-documented pattern of elevated ROS levels in CSCs, our observations demonstrated increased ROS levels, particularly mitochondrial ROS, in lung CSCs. While the present study does not elaborate on the potential relationship between high ROS levels and lung CSCs stemness, it uncovers a previously unrecognized functional role of CSC-derived ROS: specifically, their involvement in modulating T cell differentiation, which in turn contributes to the establishment of an immunosuppressive tumor microenvironment.

In the mitochondrial matrix, glutamate is converted to α-ketoglutarate (α-KG), which integrates into the tricarboxylic acid (TCA) cycle to support electron transport chain (ETC) activity and ATP generation. Glutaminolysis promotes intrinsic ROS production by enhancing TCA cycle activity and ETC flux (33) Conversely, glutamate derived from glutamine also mitigates oxidative damage by facilitating cystine uptake and glutathione (GSH) synthesis (34). While ROS accumulation has been reported in CSCs, these cells also exhibit elevated GSH activity (35). Notably, glutamine metabolism flux differs substantially among CSCs from various tumor types. In drug-resistant prostate cancer cells, increased GLS activity raises glutamate and α-KG levels, thereby significantly enhancing mitochondrial respiration (34). In gastric CSCs, upregulated GLUL boosts TCA cycle activity (36). In our study, we observed simultaneous upregulation of GLS and GLUL, accompanied by increased mitochondrial ROS levels. Our previous work revealed enhanced mitochondrial activity in NSCLC CSCs (10). Whereas the present study did not directly address this relationship, the observed elevation in mtROS from enhanced glutamine metabolism likely reflects this increased mitochondrial activity. Collectively, these results point to a role for both GLS and GLUL in driving mitochondrial ROS production.

Within the TME, Tregs are highly activated and immunosuppressive, typified by upregulated FoxP3 expression (37). Treg accumulation results from chemokine-driven recruitment (CCL17, CCL22, CCL5) of thymic and peripheral Tregs (38), as well as local expansion, differentiation and survival supported by tumor-derived TGF-β and IL-10 (39). These factors also drive conversion of naïve CD4^+^FoxP3^-^ T cells to FoxP3^+^ Tregs. Additionally, the TME promotes demethylation of the FoxP3 TSDR, which stabilizes FoxP3 expression and preserves Treg suppressive function and lineage stability (40). ROS play an essential role in Treg biology. Notably, ROS levels are higher in Tregs than in Teffs (41–43), and this elevated ROS production contributes to Treg-mediated immunosuppression, which limits anti-tumor T cell responses within the TME (42–44). Increasing ROS generation enhances Treg proliferation and survival (42, 45, 46), whereas decreased ROS levels impair Treg function and differentiation, indicating that ROS is critical for their suppressive activity (42, 43). Furthermore, Tregs exhibit greater resistance to oxidative stress-induced cell death than Teffs (46–49). Collectively, these findings establish a clear link between ROS generation and Treg differentiation. In our study, we demonstrate that the elevated ROS levels in Tregs originate from transfer by CSCs. This CSC-derived ROS promotes deubiquitination of FoxP3, thereby maintaining FoxP3 expression and enhancing Treg differentiation.

Our subsequent investigations demonstrated that NO serves as an essential intermediate in ROS-mediated regulation of FoxP3 protein abundance. Although we did not examine the mechanism by which ROS upregulates NO, previous reports suggest that ROS can activate NO synthase (NOS) (50, 51) or enhance arginine transport (52) to promote NO biosynthesis. In our study, we identified iNOS as a key effector downstream of the ROS burst. While the signaling pathways responsible for iNOS upregulation were not explored, JAK2 may be involved, as suggested by previous studies (53, 54). Furthermore, we identified FoxP3 as a substrate for S-nitrosylation. Notably, S-nitrosylation of FoxP3 appears to enhance its interaction with the deubiquitinase USP7, presumably through conformational changes in the FoxP3 protein.

In the present study, we employed human NSCLC cell lines and PDOs to investigate the effects of CSCs on T cell differentiation. These models closely recapitulate *in vivo* conditions, thereby enhancing the clinical relevance of our findings. We observed that targeting CD4^+^ T cells in a PDO-PBMC co-culture system enhanced the activation and proliferation of CD8^+^ T cells, thereby inhibiting tumor growth. However, CSCs exerted no direct effect on CD8^+^ T cell activation or proliferation, but did reduce the production of cytotoxic cytokines (**Supplementary Figures S11A–C**)—a finding consistent with previous reports.

Several limitations of this work should be acknowledged. First, the precise mechanisms underlying the enhanced glutamine metabolism in CSCs remain unclear. Second, the critical role of CSC-derived exosomal ROS in regulating FoxP3 S-nitrosylation and deubiquitination requires further validation in other human cancer types. Finally, our current translational approach—using MSX, BPTES, or L-NAME-pretreated PDOs—does not allow us to specifically isolate the role of CSCs in modulating T cell differentiation and function.

## Conclusions

In essence, CSCs exhibit increased glutamine metabolism, leading to elevated intracellular ROS levels. These ROS are encapsulated in CSC-derived exosomes, which are then transferred to T cells; this process effectively induces NO synthesis and FoxP3 S-nitrosylation. Notably, FoxP3 S-nitrosylation inhibits its degradation, thereby promoting the generation of Treg cells. Consequently, targeting cancer-associated glutamine metabolism represents a promising strategy to alleviate the immunosuppressive TME. Collectively, these findings uncover previously unrecognized aspects of CSCs and offer novel insights for therapeutic exploration in cancer treatment.

## Data Availability

The original contributions presented in the study are included in the article/**Supplementary Material**. Further inquiries can be directed to the corresponding author.
