# Supplementary material for: Cancer stem cell programs S-nitrosylation and deubiquitination of FoxP3 protein to promote regulatory T cell differentiation
Source: Front Immunol. 2026 Sep 15;17:1961819. doi: 10.3389/fimmu.2026.1961819 (PMC13619378; doi:10.3389/fimmu.2026.1961819)
Supplement: Supplementary file 1 [file DataSheet1.pdf]

Supplementary Figure 1

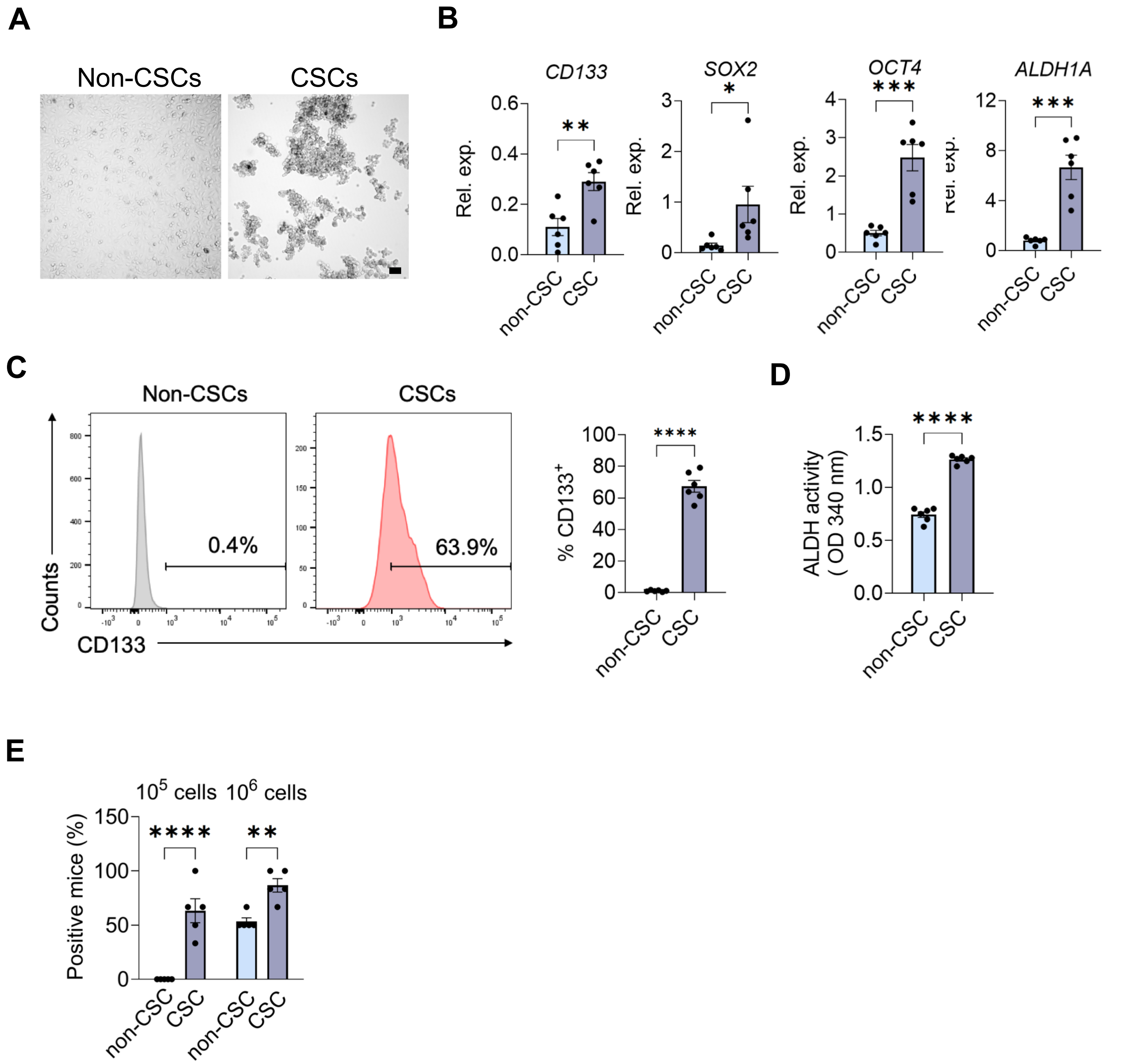

Fig.S1 CSCs and non-CSCs

(A) Representative image of CSCs (spheres) and non-CSCs (adherent cells) cultured from human lung adenocarcinoma A549 cells. (B) Detection of the indicated stem-related genes in CSCs and non-CSCs using qPCR. Mean ± SEM from 6 independent experiments. (C) The frequency of CD133-expressing fractions in CSCs (spheres) and non-CSCs (adherent cells). Mean ± SEM from 6 independent experiments. (D) The ALDH activity of CSCs and non-CSCs. Mean ± SEM from 6 independent experiments. (E) The indicated cell number of CSCs and non-CSCs were injected subcutaneously into NSG mice and tested for the positive frequency of tumorigenicity. Mean ± SEM from 5 independent experiments. *$p < 0.05$, **$p < 0.01$, ***$p < 0.001$ and ****$p < 0.0001$ with unpaired t-tests (B-D) or ANOVA with Tukey's method (E).

Supplementary Figure 2

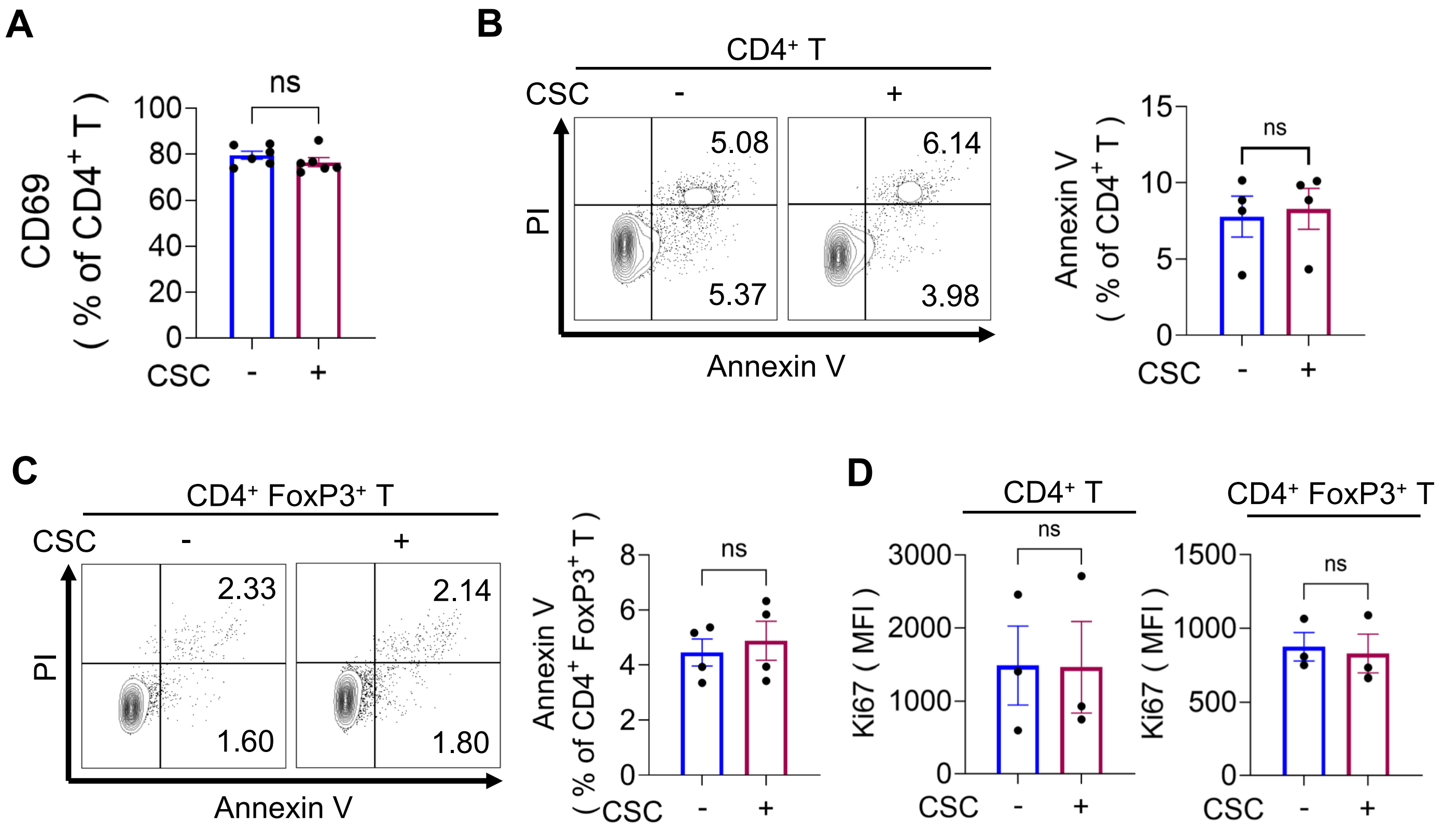

Fig.S2 CD4⁺ T cell activation, proliferation, and apoptosis are unaltered by CSCs

(A) Healthy CD4⁺ T cells were pre-conditioned with or without CSCs for 12 hours, activated with anti-CD3/CD28 beads for 24 hours, and tested for cell activation by analyzing CD69 frequencies. Mean ± SEM from 6 individuals in each group. (B-D) Healthy CD4⁺ T cells were pre-incubated with or without CSCs for 12 hours, then activated with anti-CD3/CD28 beads for 72 hours. Apoptosis in total CD4⁺ T cells and FoxP3⁺ Treg cells was assessed by Annexin V/PI staining, and proliferation was evaluated by Ki67 expression via flow cytometry. Data are shown as mean ± SEM from 3–4 independent donors per group.

Supplementary Figure 3

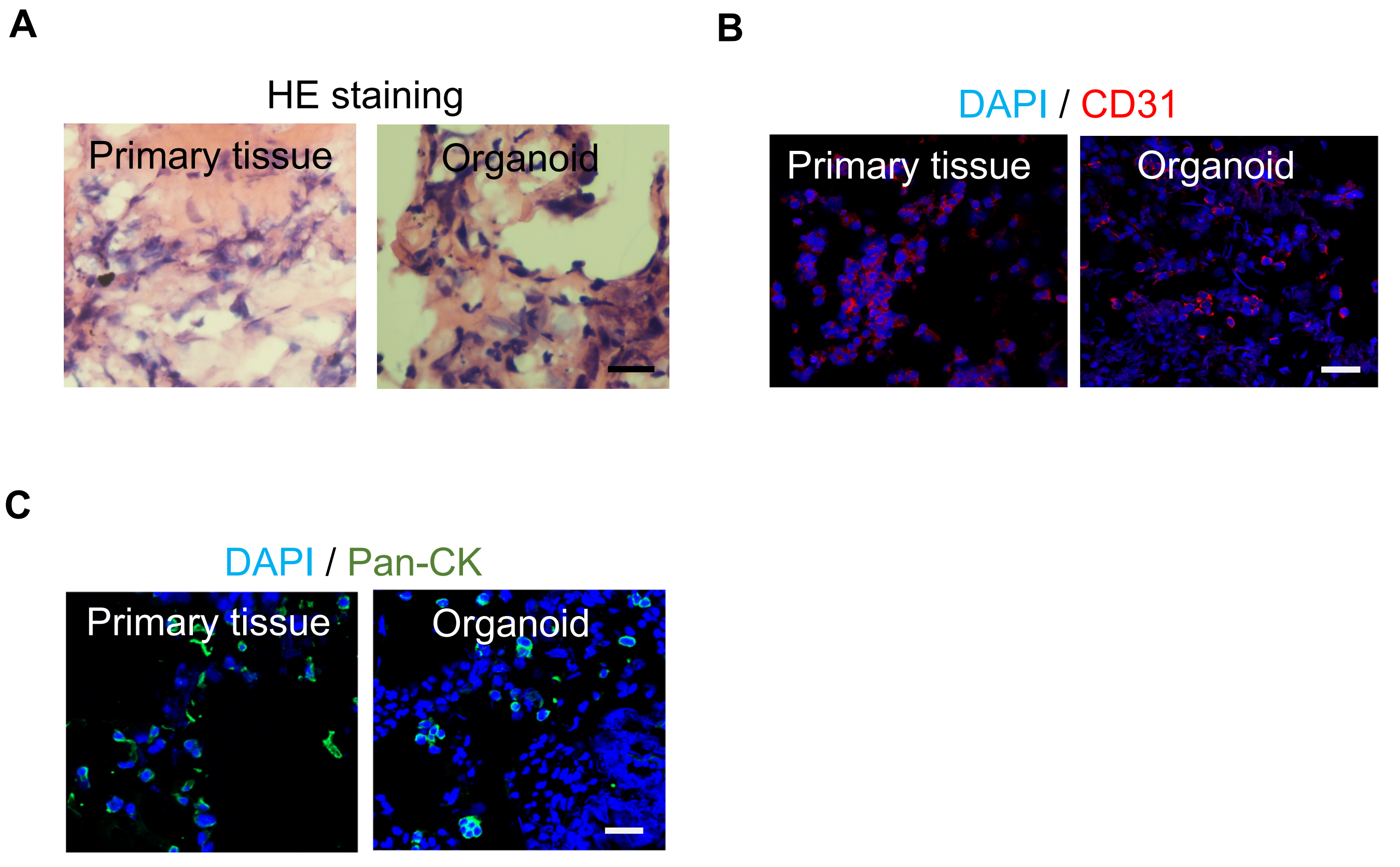

Fig.S3 Establishment of NSCLC PDOs

(A) Representative HE staining images from PDO and matched primary tumor tissue. Scale bar: 100 µm. (B) Representative immunofluorescence micrographs of paired PDO and primary tumor sample immunostained for CD31, with Hoechst used for nuclear counterstaining. Scale bar: 20 µm. (C) Representative immunofluorescence images of PDO-primary tumor pair stained for Pan-Ck; nuclei were counterstained using Hoechst. Scale bar: 20 µm.

Supplementary Figure 4

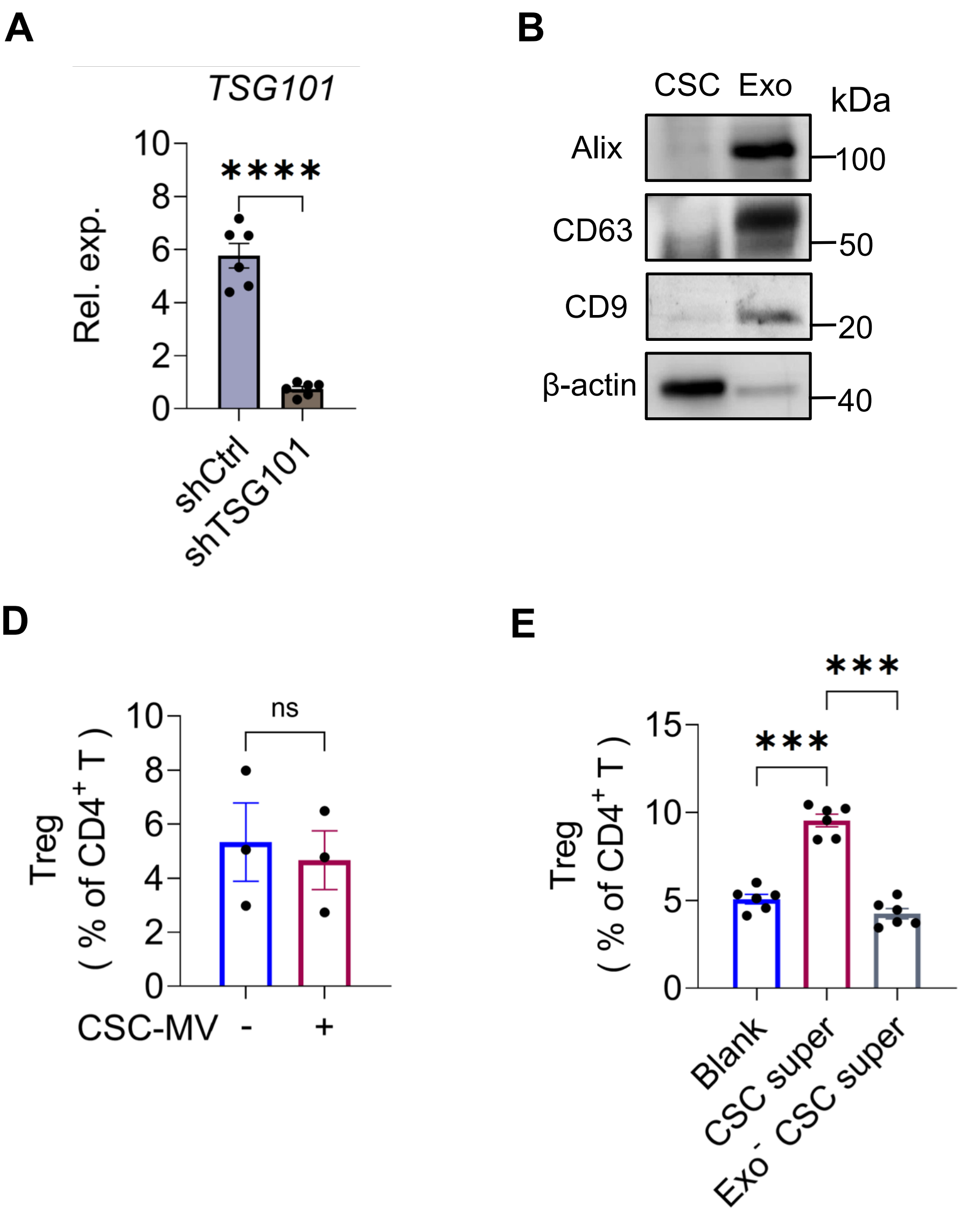

Fig.S4 Exosomes, but not MVs, mediate the effects of CSCs on CD4⁺ T cells

(A) CSCs were transfected with TSG101 shRNA or control shRNA for 12 hours, and knockdown efficiency was determined by qPCR. Data are presented as mean ± SEM from 6 independent experiments. (B) The identity of CSC-derived exosomes was verified by immunoblot analysis of the indicated marker proteins. (C-D) Healthy $CD4^+$ T cells were treated with CSC-derived MVs for 12 hours, then activated with anti-CD3/CD28 beads for 4 days. The frequencies of T-bet^+ Th1 and $FoxP3^+$ Treg cells were determined by flow cytometry. Data represent mean ± SEM from 3 independent donors. (E) Healthy $CD4^+$ T cells were treated with CSC-derived supernatant or exosome-depleted supernatant for 12 hours, followed by 4-day activation with anti-CD3/CD28 beads. The frequency of Treg cells was determined by flow cytometry. Data are shown as mean ± SEM from 6 independent donors. ***p < 0.001, ****p < 0.0001, as determined by paired t-test (A) or one-way ANOVA with Tukey's method (E).

Supplementary Figure 5

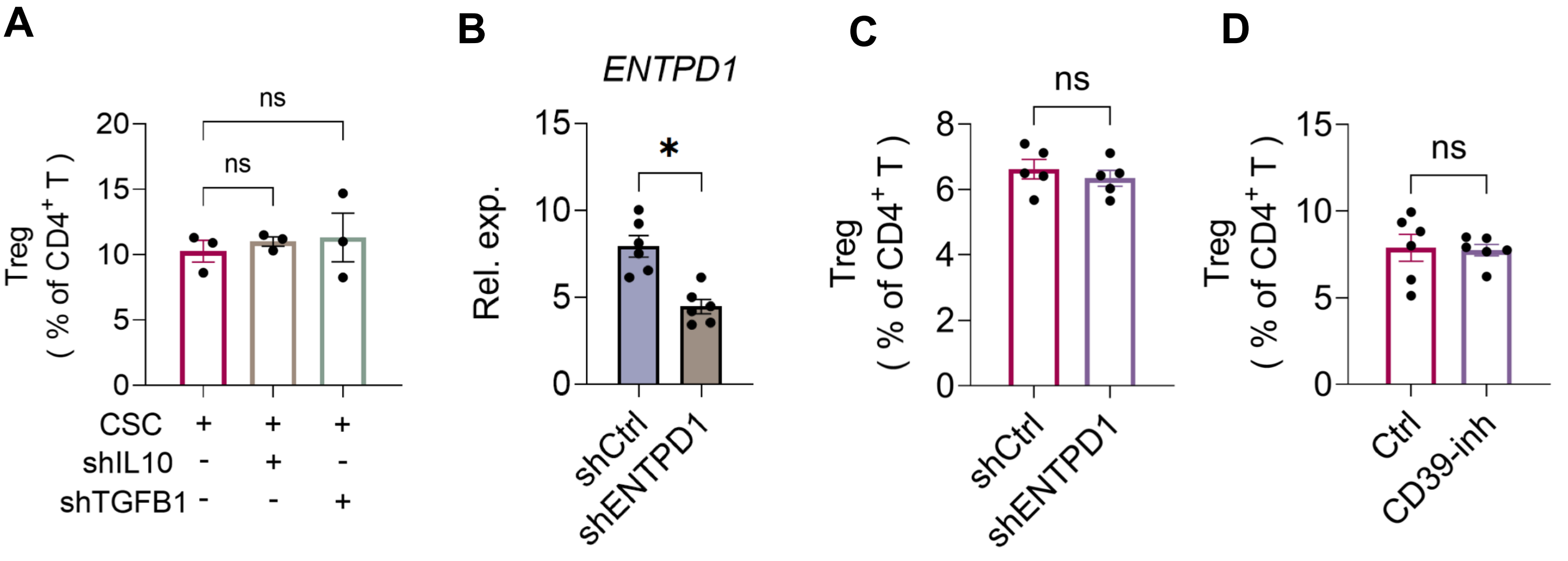

Fig.S5 IL-10, TGF-β, and CD39 are not responsible for CSC-induced enhancement of Treg differentiation

(A) CSCs were transfected with shRNA targeting IL10 or TGFB1 or control shRNA for 24 hours, then co-incubated with healthy $CD4^+$ T cells for 12 hours, followed by T cell activation with anti-CD3/CD28 beads for 4 days. The frequency of $FoxP3^+$ Treg cells was determined by flow cytometry. Data are shown as mean ± SEM from 3 independent donors. (B) CSCs were transfected with shRNA targeting human CD39 or control shRNA for 24 hours, and knockdown efficiency was assessed by qRT-PCR at 12 hours post-transfection. Data are shown as mean ± SEM from 6 independent experiments. (C) CSCs were transfected with CD39 shRNA or control shRNA for 24 hours, then co-incubated with healthy $CD4^+$ T cells for 12 hours, followed by T cell activation with anti-CD3/CD28 beads for 4 days. The frequency of $FoxP3^+$ Treg cells was determined by flow cytometry. Data are shown as mean ± SEM from 5 independent donors. (D) CSCs were pretreated with the CD39 inhibitor ARL67156 (10 μM) for 24 hours prior to co-culture with $CD4^+$ T cells, followed by T cell activation for 4 days. Treg frequency was determined by flow cytometry. Data are shown as mean ± SEM from 6 independent donors. *$p < 0.05$ with paired t-tests (B).

Supplementary Figure 6

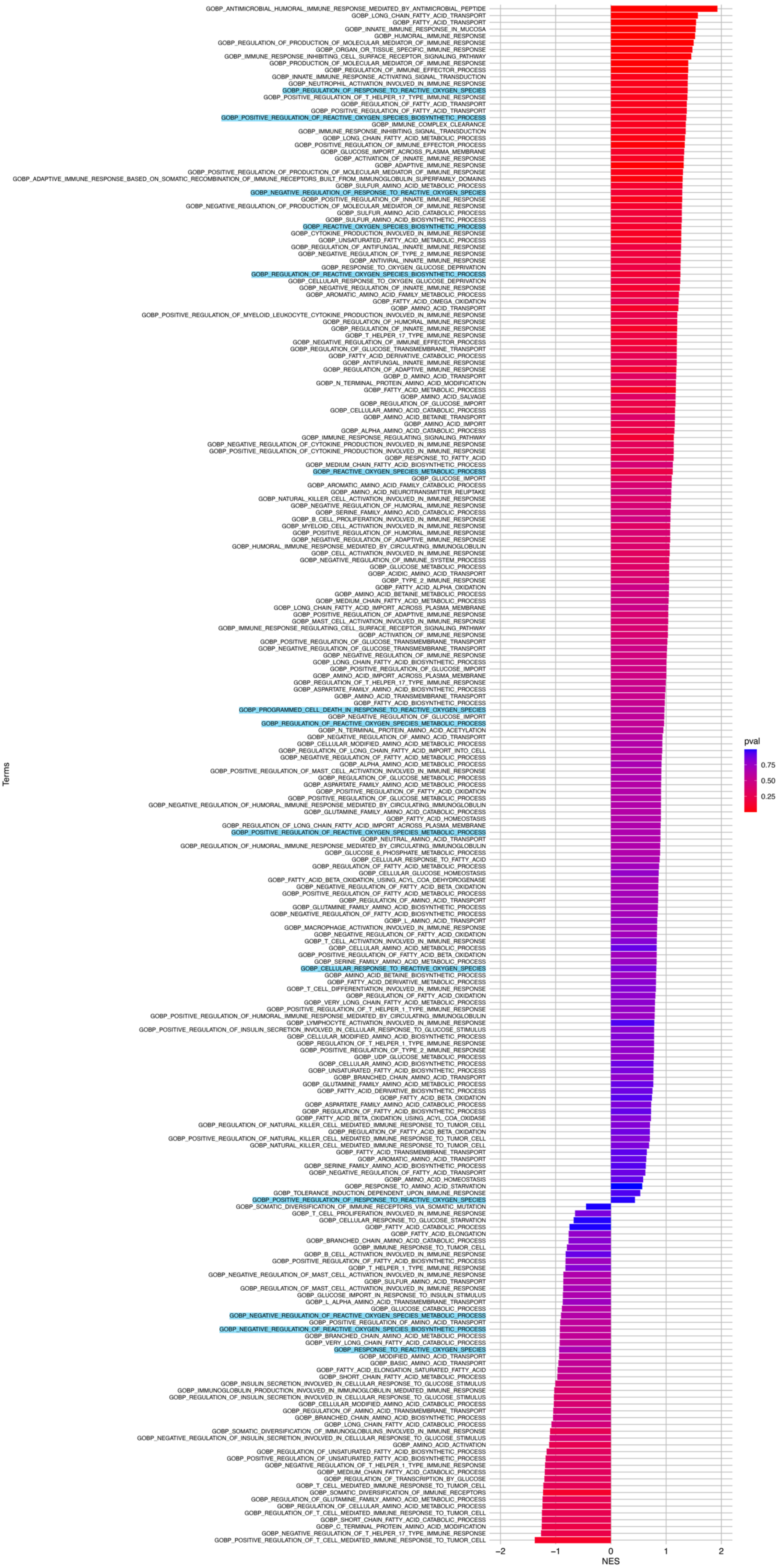

Fig.S6 RNA sequencing of CSCs and non-CSCs

Bulk RNA-seq revealed distinct pathway alterations in CSCs versus non-CSCs, with ROS-associated pathways being particularly prominent.

Supplementary Figure 7

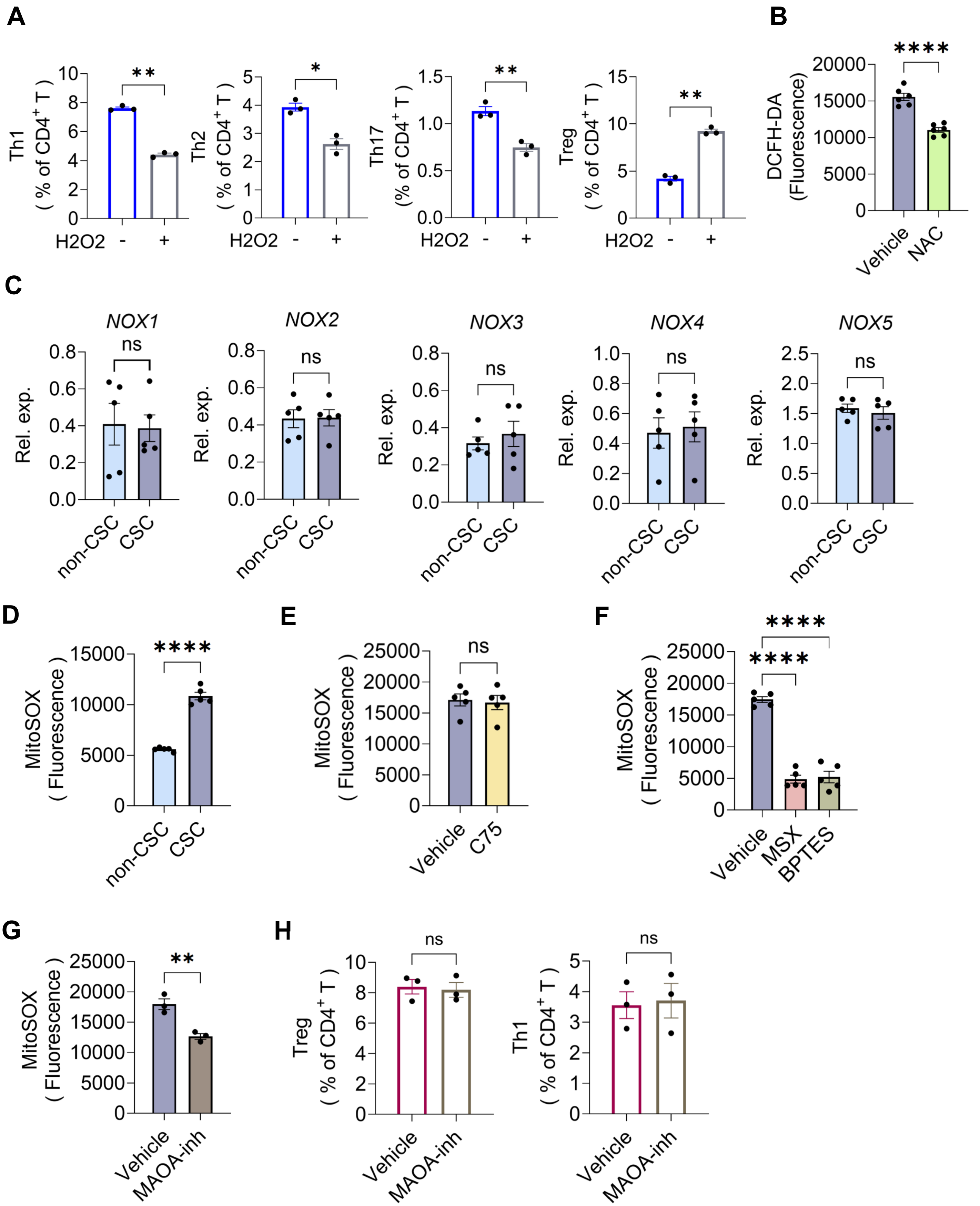

Fig.S7 Glutamine metabolism-derived ROS are the primary drivers of CSC-induced Th cell mal-differentiation

(A) Healthy $CD4^+$ T cells were stimulated with anti-CD3/CD28 beads for 4 days in the presence or absence of H2O2 (10 μM). The frequencies of T-bet^+ Th1, $GATA3^+$ Th2, $ROR\gamma t^+$ Th17, and $FoxP3^+$ Treg cells were determined by flow cytometry. Data are shown as mean ± SEM from 3 independent donors per group. (B) CSCs were treated with or without the ROS inhibitor NAC (10 μM) for 12 hours, and intracellular ROS levels were measured using DCFH-DA. Data are shown as mean ± SEM from 6 independent experiments. (C) The mRNA expression levels of the indicated genes involved in ROS generation were examined in CSCs and non-CSCs by qRT-PCR. Data are shown as mean ± SEM from 5 independent experiments. (D) Mitochondrial ROS levels in CSCs and non-CSCs were analyzed using MitoSOX fluorescence. Data are shown as mean ± SEM from 5 independent experiments. (E) CSCs were treated with or without the FASN inhibitor C75 (10 μM) for 24 hours, and mitochondrial ROS levels were detected. Mean ± SEM from 5 independent experiments. (F) CSCs were treated with or without MSX (1 mM) or BPTES (1 μM) for 12 hours, and the indicated ROS levels were detected. Mean ± SEM from 5 independent experiments. (G) CSCs were treated with or without the MAOA inhibitor MAO-IN-M30 (50 nM) for 24 hours, and mitochondrial ROS levels were measured. Data are shown as mean ± SEM from 3 independent experiments. (H) CSCs were treated with or without MAO-IN-M30 (50 nM) for 24 hours, then co-incubated with healthy $CD4^+$ T cells for 12 hours, followed by T cell activation with anti-CD3/CD28 beads for 4 days. The frequencies of T-bet^+ Th1 and $FoxP3^+$ Treg cells were determined by flow cytometry. Data are shown as mean ± SEM from 3 independent donors per group. $^*p < 0.05$, $^{**} p < 0.01$, and $^{****}p < 0.0001$ with paired (A-B, G) and unpaired (D) t-tests or ANOVA with Tukey's method (F).

Supplementary Figure 8

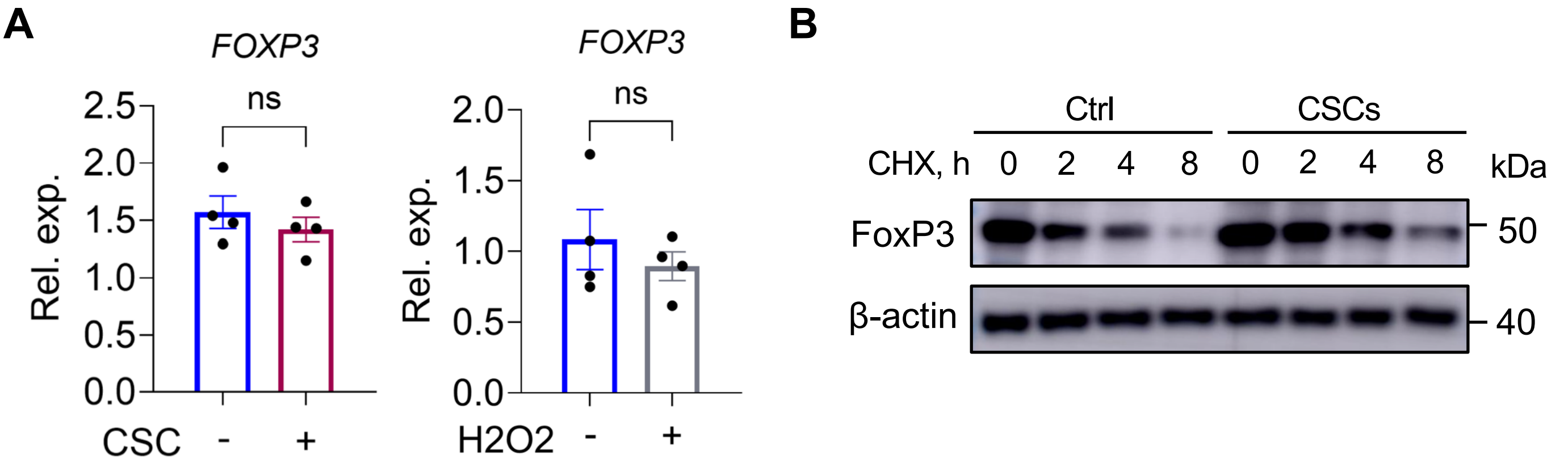

Fig.S8 CSCs enhance the stability of FoxP3 in T cells

(A) Healthy CD4$^+$ T cells were pre-conditioned with CSCs for 12 hours, then activated with anti-CD3/CD28 beads for 4 days. The mRNA expression levels of FoxP3 were determined by qPCR. Data are shown as mean ± SEM from 4 independent donors. (B) Healthy CD4$^+$ T cells were pre-conditioned with CSCs for 12 hours, then activated with anti-CD3/CD28 beads for 4 days. Treg cells were subsequently treated with cycloheximide (CHX, 20 μg/mL) for the indicated time points, and FoxP3 protein levels were analyzed by immunoblotting.

Supplementary Figure 9

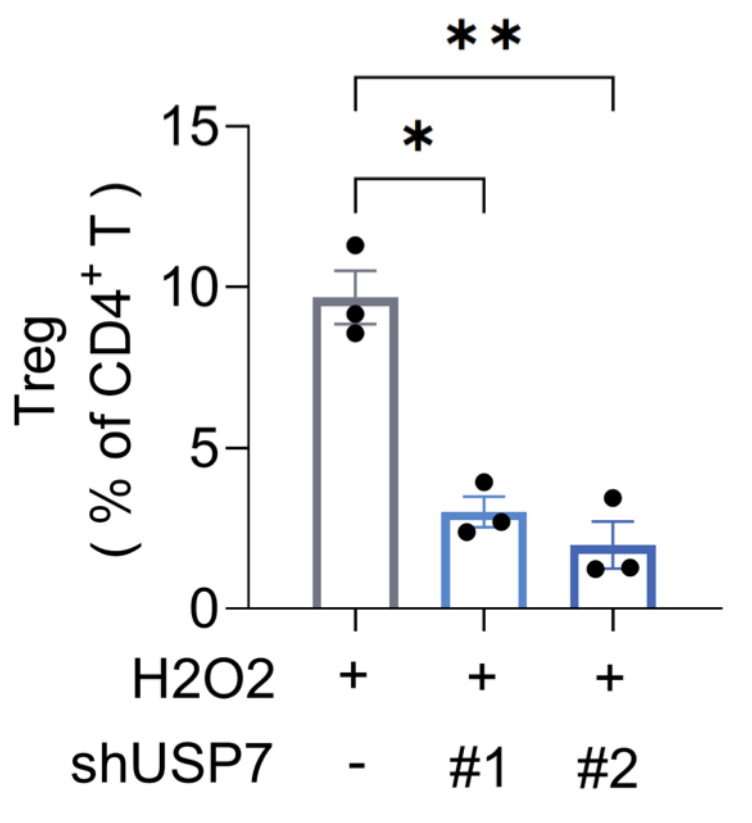

Fig.S9 USP7 knockdown rescues H2O2-enhanced Treg differentiation

(A) Healthy $CD4^+$ T cells were activated with anti-CD3/CD28 beads for 4 days in the presence or absence of H_2O_2 (10 μM), and the frequency of $FoxP3^+$ Treg cells was determined by flow cytometry. Data are shown as mean ± SEM from 3 independent donors. *$p < 0.05$, **$p < 0.01$ by ANOVA with Tukey's method.

Supplementary Figure 10

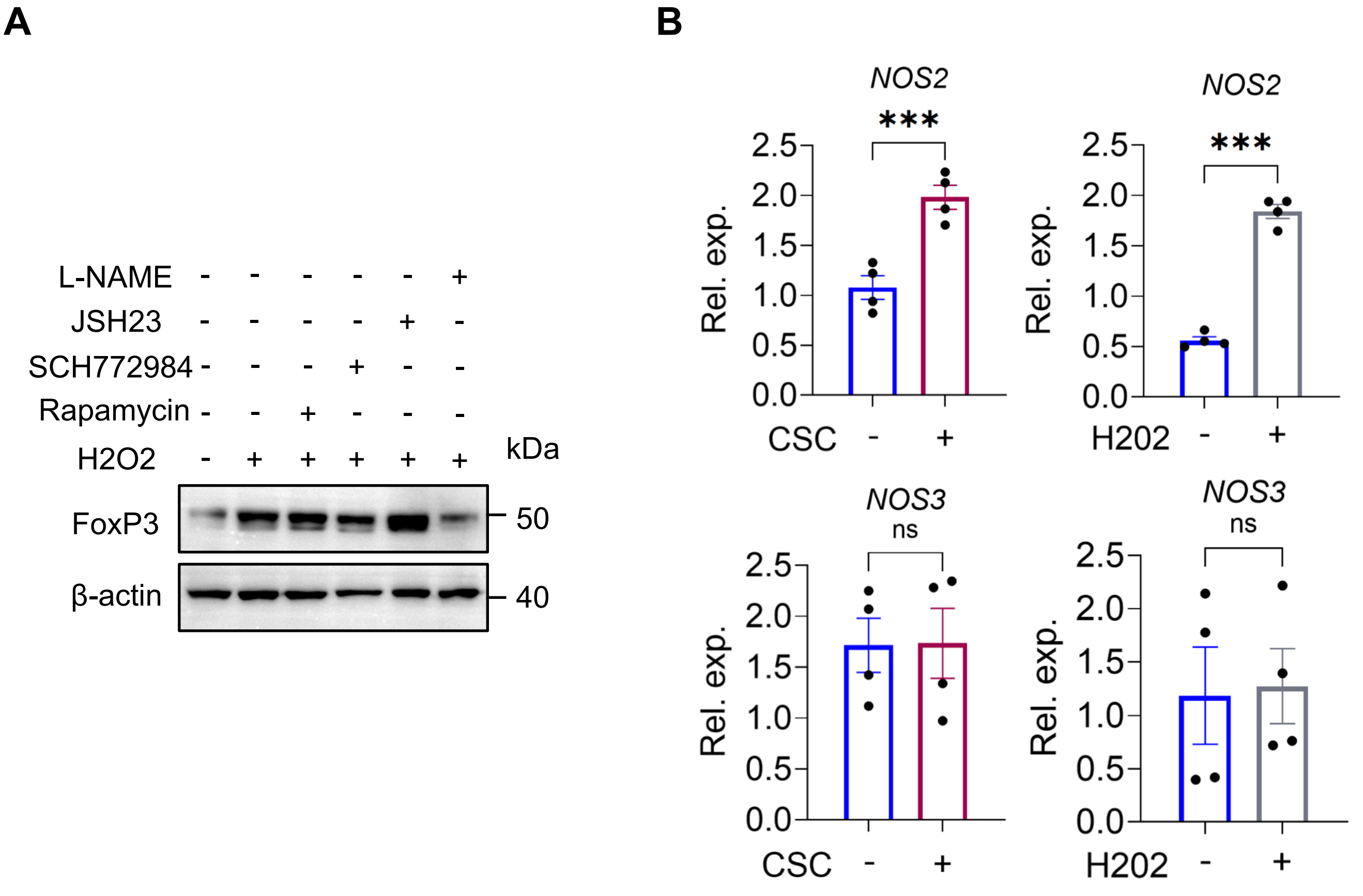

Fig.S10 CSCs imprint the NO signaling pathway in CD4⁺ T cells

(A) Healthy CD4⁺ T cells were activated for 4 days using anti-CD3/CD28 beads, with or without H2O2, in the absence or presence of Rapamycin (0.1 nM), SCH772984 (1 nM), JSH-23 (10 μM), or L-NAME (100 μM). Western blot analysis was subsequently performed to examine the protein levels of FoxP3. (B) CD4⁺ T cells were pre-conditioned ± CSCs for 12 h, then activated for 4 days ± H2O2. NOS2 and NOS3 mRNA levels were measured by qRT-PCR. Data are shown as mean ± SEM from 4 independent donors. ****p* < 0.001 with paired t-test (B).

Supplementary Figure 11

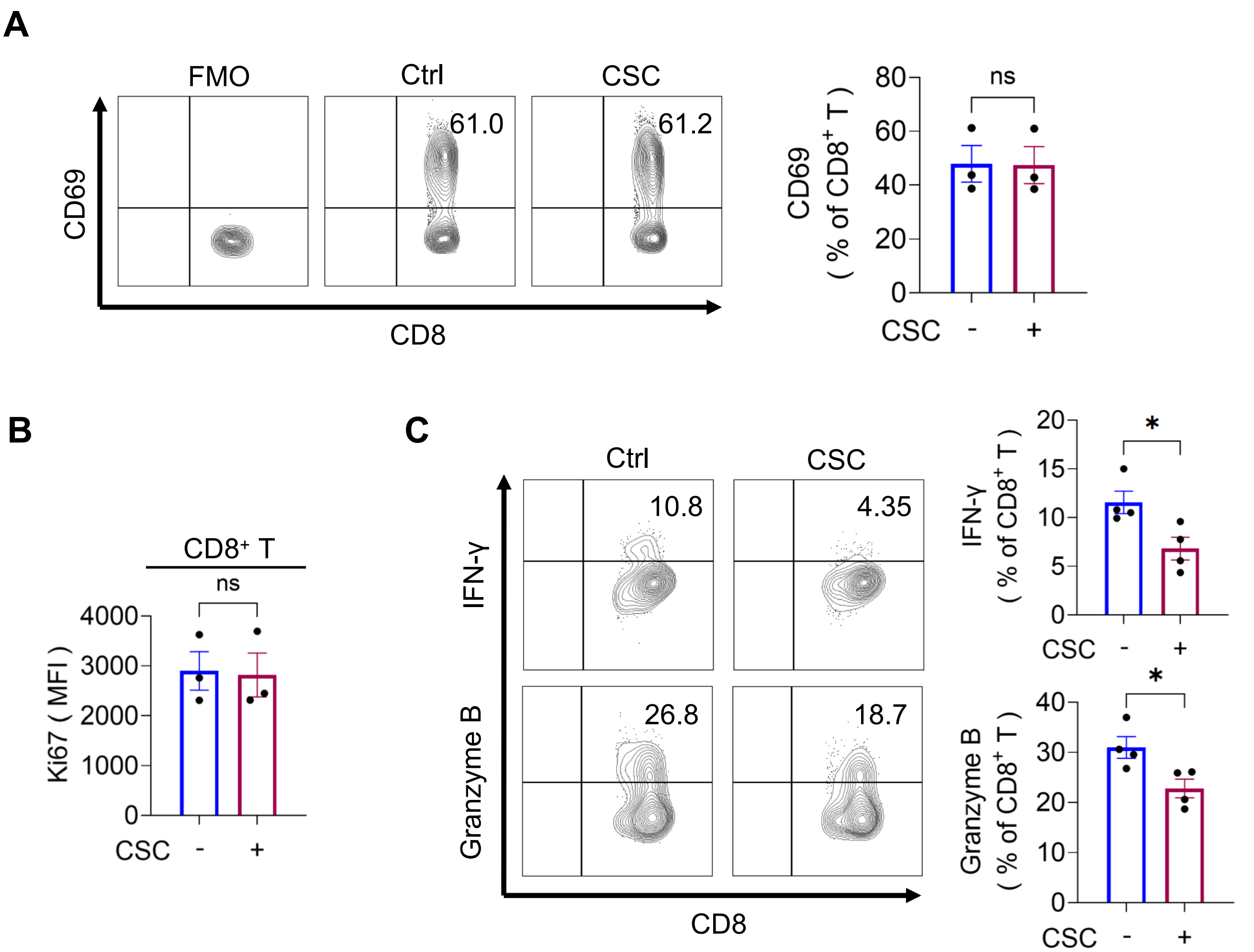

Fig.S11 CSCs impair cytokine secretion by CD8⁺ T cells without affecting activation or proliferation

(A) Healthy $CD8^+$ T cells were pre-conditioned with or without CSCs for 12 hours, then activated with anti-CD3/CD28 beads for 24 hours. T cell activation was assessed by measuring the frequency of $CD69^+$ cells via flow cytometry. Data are shown as mean ± SEM from 3 independent donors per group. (B) Healthy $CD8^+$ T cells were pre-incubated with or without CSCs for 12 hours, then activated with anti-CD3/CD28 beads for 72 hours. Proliferation was evaluated by Ki67 expression using flow cytometry. Data are shown as mean ± SEM from 3 independent donors per group. (C) Healthy $CD8^+$ T cells were pre-incubated with or without CSCs for 12 hours, then activated with anti-CD3/CD28 beads for 4 days. Intracellular IFN-γ and Granzyme B expression were determined by flow cytometry. Data are shown as mean ± SEM from 4 independent donors per group. *$p < 0.05$ with paired t-test (C).
